# Implications of different cell death patterns for prognosis and immunity in lung adenocarcinoma

**DOI:** 10.1038/s41698-023-00456-y

**Published:** 2023-11-15

**Authors:** Yang Zhou, Weitong Gao, Yu Xu, Jiale Wang, Xueying Wang, Liying Shan, Lijuan Du, Qingyu Sun, Hongyan Li, Fang Liu

**Affiliations:** 1 Department of Medical Oncology, Harbin Medical University Cancer Hospital, 150081 Harbin, China; 2 College of Resources and Environment, Northeast Agricultural University, 150030 Harbin, China; 3 Department of Otolaryngology Head and Neck Surgery, Xiangya Hospital, Central South University, 410008 Changsha, China

**Keywords:** Non-small-cell lung cancer, Immunosurveillance

## Abstract

In recent years, lung adenocarcinoma (LUAD) has become a focus of attention due to its low response to treatment, poor prognosis, and lack of reliable indicators to predict the progression or therapeutic effect of LUAD. Different cell death patterns play a crucial role in tumor development and are promising for predicting LUAD prognosis. From the TCGA and GEO databases, we obtained bulk transcriptomes, single-cell transcriptomes, and clinical information. Genes in 15 types of cell death were analyzed for cell death index (CDI) signature establishment. The CDI signature using necroptosis + immunologic cell death-related genes was established in the TCGA cohort with the 1-, 2-, 3-, 4- and 5-year AUC values were 0.772, 0.736, 0.723, 0.795, and 0.743, respectively. The prognosis was significantly better in the low CDI group than in the high CDI group. We also investigated the relationship between the CDI signature and clinical variables, published prognosis biomarkers, immune cell infiltration, functional enrichment pathways, and immunity biomarkers. In vitro assay showed that HNRNPF and FGF2 promoted lung cancer cell proliferation and migration and were also involved in cell death. Therefore, as a robust prognosis biomarker, CDI signatures can screen for patients who might benefit from immunotherapy and improve diagnostic accuracy and LUAD patient outcomes.

## Introduction

Globally, as the most common malignancy, lung cancer causes the most deaths from cancer^1,2^. Lung cancer caused by NSCLC comprises nearly 80% of cases, while lung adenocarcinoma (LUAD) accounts for ~50%. Patients with LUAD have experienced significant improvements in their clinical outcomes thanks to advances in surgery, molecular-targeted therapy, and immunotherapy in recent years. However, it remains rare for patients with LUAD to survive 5 years after diagnosis^3–6^. Therefore, the development of predictive models for LUAD detection, prognosis, and immunotherapy is therefore urgently needed.

Due to the inborn nature of most tumors and their resistance to apoptosis, inducing cell death pathways, such as necroptosis and immunogenic cell death has gradually become a potential therapeutic means^7,8^. As a form of programmed cell death, necroptosis enhances CD8^+^ T cell-mediated antitumor immunity via RIPK3 and RIPK1 activation^9^. Moreover, necroptosis can also modulate the tumor’s immune microenvironment by altering the expression of immune checkpoints^10^. Therefore, necroptosis has been promised to be a novel target for immunotherapy in LUAD. Furthermore, the contribution of necroptosis to cancer prognosis has been explored in depth in recent studies. When RIPK1, RIPK3, and MLKL are knocked down in colon and esophageal cancers, tumor growth is inhibited by the reduction of NF-κB activity^11^. Another type of cell death, immunogenic cell death (ICD) has been characterized by the release of molecular signals, referred to as damage-associated molecular patterns (DAMPs) including cell surface-mainly calreticulin (CRT), adenosine triphosphate (ATP) and high mobility group box 1 protein (HMGB1)^12,13^. The above DAMPs bind to specific receptors on the surface of dendritic cells to induce the anti-tumor immune response. Meanwhile, the latest study demonstrated that eIF2α phosphorylation correlated with CRT exposure and constitutes a pathognomonic characteristic of ICD^14,15^.

Some necroptosis and ICD-related genes have recently been regarded as prognostic markers for cancer^11,16,17^. Recently, the necroptosis-related gene IGF2BP1 was reported to play crucial roles in the progression and prognosis of lung cancer. In LUAD patients with high expression of IGF2PB1, the clinical stage and prognosis were significantly worse^18^. Mechanically, TFAP4 activated IGF2BP1 from its transcriptional level, which promoted proliferation, migration, and invasion of NSCLC cells via m6A modification^19^. Besides, IGF2BP1-phase separation is associated with c-myc-mediated progression and proliferation in lung cancer cells mediated by MNX1-AS1, implying the MNX1-AS1/IGF2BP1 axis may serve as a potential biomarker and therapeutic target in NSCLC^20^. ICD-related gene toll-like receptor 2 (TLR2), a regulator of oncogene-induced senescence, is an important tumor suppressor in premalignancy. A recent study has demonstrated that early TLR2 activation correlates with improved survival and clinical regression in lung tumorigenesis due to the activation of cell cycle arrest pathways and proinflammatory phenotype^21^. And TLR2/MYD88 axis signaling induces arginase1 mRNA expression in tumor-related neutrophils in NSCLC, highlighting the critical role that neutrophil cells play in the suppression of lymphocytes infiltrated in tumors^22^. In spite of this, it remains unclear how the abovementioned genes contribute to LUAD. Thus, a better understanding of necroptosis and ICD-related genes helps investigate potential biomarkers and guide immunotherapeutic interventions in LUAD.

In our study, for training and validation cohorts, we collected 439 patients from the TCGA, 196 patients from GSE37745, 442 patients from GSE68465, 27 patients from GSE135222, 348 patients from IMvigor210 and 27 patients for GSE78220. For single-cell RNA transcriptome datasets, we collected 9 samples of 8 patients from GSE171145. We incorporated necroptosis and ICD-related genes and selected the genes that were tightly related to LUAD survival and prognosis. Then we established the CDI signature based on the above genes and further explored the difference in prognosis, immune infiltration, and signaling pathway in high and low CDI groups, offering insights into prognosis prediction and immune landscape in LUAD (Supplementary Fig. 1).

## Results

### The relationship of cell death-related genes with LUAD prognosis

**Fig. 1:**
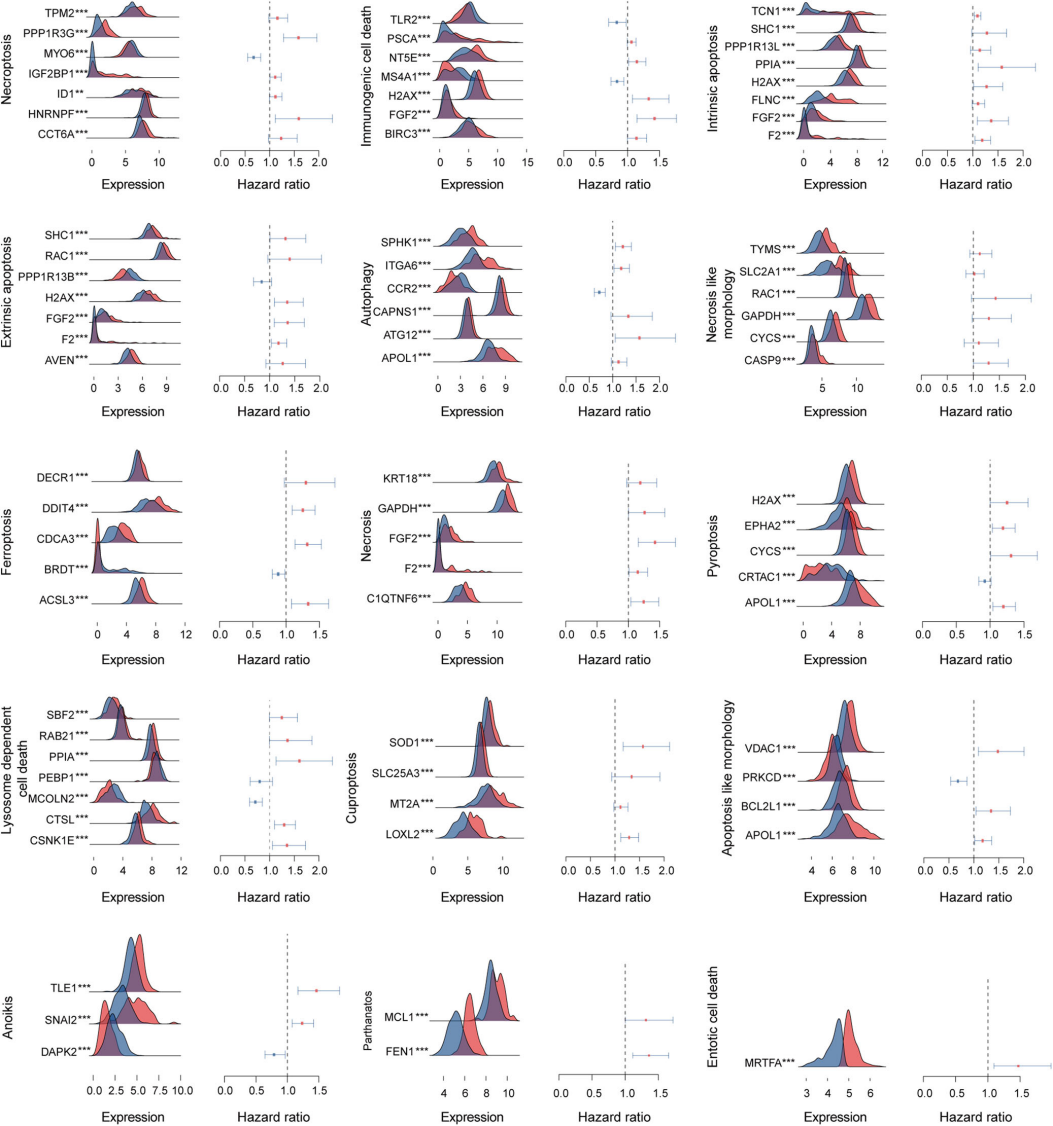
The 15 types of cell death-related gene expression in high and low-risk groups and associations of cell death-related genes with LUAD prognosis using multivariate Cox regression. Red represented the high-risk group while blue represented the low-risk group. *P < 0.05, **P < 0.01, ***P < 0.001.

We investigated the genes which were significantly related with LUAD prognosis and then analyzed the differential expression of cell death-related genes in Fig. 1. Forest plots using multivariate analysis showed that necroptosis-related genes including CCT6A (HR = 1.233, 95% CI = 0.975–1.560, *P* = 0.081), HNRNPF (HR = 1.593, 95% CI = 1.115–2.277, *P* = 0.011), ID1 (HR = 1.117, 95% CI = 0.996–1.252, *P* = 0.058), IGF2BP1 (HR = 1.113, 95% CI = 1.005−1.233, *P* = 0.040), MYO6 (HR = 0.671, 95% CI = 0.551−0.817, *P* = 7.22E−05), PPP1R3G (HR = 1.586, 95% CI = 1.283−1.960, *P* = 1.97E−05), TPM2 (HR = 1.157, 95% CI = 0.980–1.365, *P* = 0.085) in which HNRNPF, IGF2BP1, MYO6, and PPP1R3G were independent predictors of OS. Forest plots using multivariate analysis also showed that immunologic cell death-related genes including BIRC (HR = 1.138, 95% CI = 0.997–1.299, *P* = 0.055), FGF2 (HR = 1.422, 95% CI = 1.146–1.765, *P* = 0.001), H2AX (HR = 1.331, 95% CI = 1.076−1.647, *P* = 0.006), MS4A1 (HR = 0.834, 95% CI = 0.739−0.941, *P* = 0.003), NT5E (HR = 1.144, 95% CI = 1.017−1.285, *P* = 0.024), PSCA (HR = 1.060, 95% CI = 0.993–1.133, *P* = 0.081), TLR2 (HR = 0.829, 95% CI = 0.703−0.977, *P* = 0.025) in which FGF2, H2AX, MS4A1, NT5E, TLR2 were independent predictors of OS. Besides, the relationship of other cell death types related genes with LUAD OS was shown in Supplementary file 1. Then, we established the necroptosis-related risk score model and immunologic cell death-related risk score model using genes significantly linked to LUAD prognosis based on the following formula, respectively: The necroptosis-related risk score = CCT6A∗0.20954219 + HNRNPF∗0.465856898 + ID1∗0.11066557 + IGF2BP1∗0.106862091−MYO6∗0.399018963 + PPP1R3G∗0.461256604 + TPM2∗0.145578455 and the immunologic cell death-related risk score = BIRC3∗0.129259936 + FGF2∗0.352407793 + H2AX∗0.2859114−MS4A1∗0.181392849 + NT5E∗0.134192126 + PSCA∗0.058592815−TLR2∗0.187882913. Other cell death types related risk score was also calculated based on the same formula in Supplementary file 2. Based on the best cut-off values of risk score, we divided patients into high and low-risk groups. The differential expression of cell death-related genes between high and low-risk groups was also shown in Fig. 1. For example, necroptosis-related genes including TPM2, PPP1R3G, IGF2BP1, ID1, HNRNPF, and CCT6A as well as immunologic cell death-related genes including PSCA, NT5E, H2AX, FGF2, BIRC3 were significantly expressed in high-risk groups indicating these genes play potentially critical roles in cancer development and progression.

### The establishment and validation of cell death index (CDI) signature

**Fig. 2:**
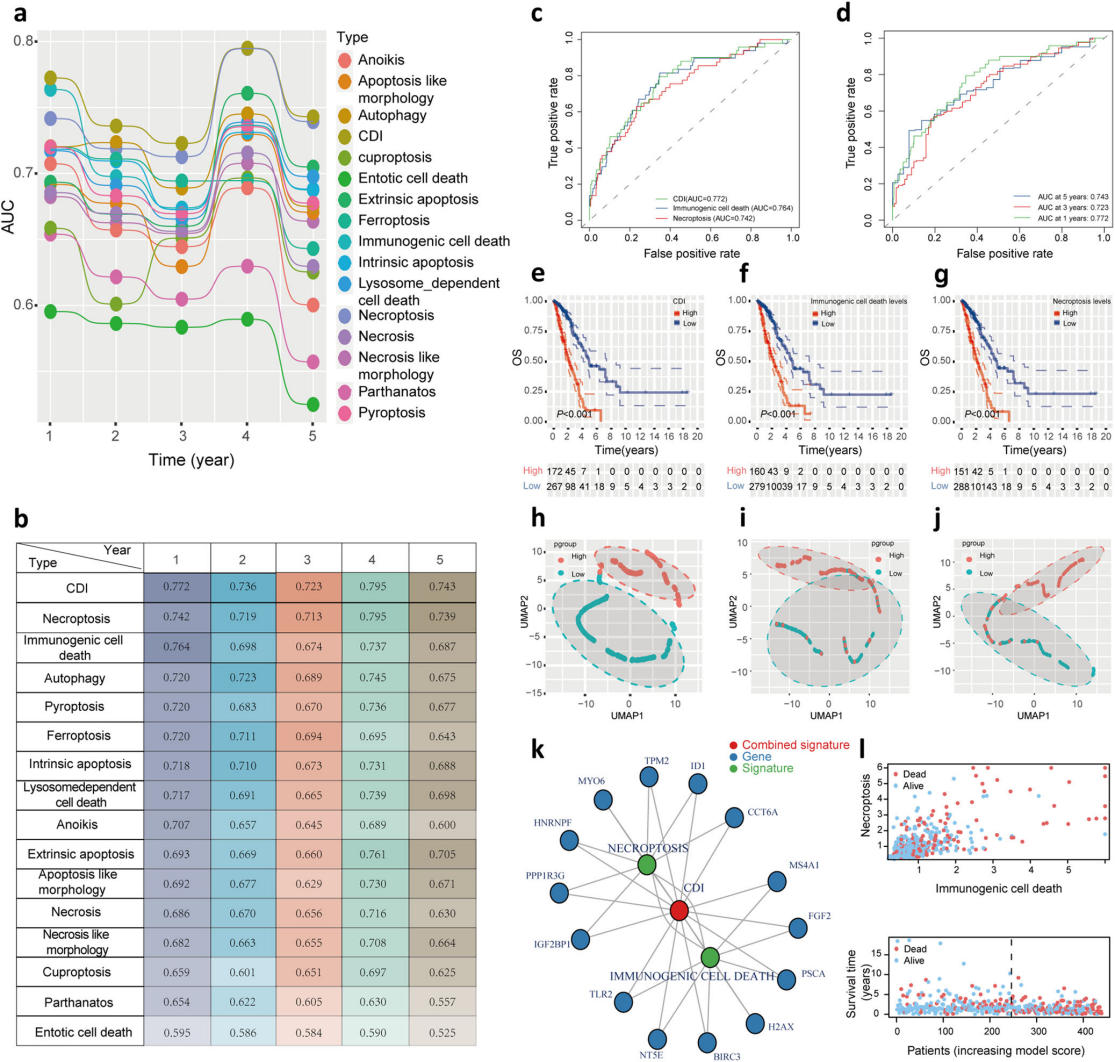
The prognosis value of CDI signature in LUAD. **a**, **b** A comparison of 1-, 2-, 3-, 4-, and 5-year AUC values among 15 types of cell death signature showed the superiority of CDI signature. **c** A comparison of ROC curve in CDI signature with necroptosis and immunologic cell death signature. **d** The 1-, 3-, and 5-year ROC curve of CDI signature suggested that all AUC values were over 0.70. **e–g** The Kaplan–Meier survival curve with log-rank test demonstrated the relationship between OS and CDI signature, necroptosis, and immunologic cell death signature, respectively. **h–j** Clustering analysis showed gene classification in high and low-risk groups based on CDI signature, necroptosis, and immunologic cell death signature, respectively. **k** Diagram of gene interaction network showed that CDI signature was tightly associated with necroptosis and immunologic cell death-related genes. **l** The distribution of survival status based on necroptosis, immunologic cell death signature and CDI signature.

According to Fig. 2a, b, the 1-, 2-, 3-, 4-, and 5-year AUC values for 15 types of cell death signatures have been calculated and compared. To explore the combined cell death-related signatures with the highest AUC values, we evaluated a series of candidate GLMs that were composed of different combinations of cell death types. It was found that the necroptosis + immunologic cell death signature, which we named the cell death index (CDI) signature, had higher predictive performance. The 1-, 2-, 3-, 4-, and 5-year AUC values were 0.772, 0.736, 0.723, 0.795, and 0.743, respectively (Fig. 2b, d). The ROC curve also supported the above conclusion that the 1-year AUC value of CDI signature (0.772) was higher than that of necroptosis or immunologic cell death signature (0.764 and 0.742) in Fig. 2c. Based on CDI, necroptosis, and immunologic cell death levels, we classified LUAD patients into high and low-risk groups. The log-rank test was used to further demonstrate the difference between high and low-risk groups for LUAD survival. It has been shown that LUAD patients at low risk have a significantly longer overall survival than those at high risk (*P* < 0.05) according to Kaplan–Meier survival curves (Fig. 2e–g). A uniform manifold approximation and projection (UMAP) also revealed the classification of genes into high- and low-risk groups in Fig. 2h–j. In the gene interaction network, CDI signature was closely associated with genes related to necroptosis and immunologic cell death genes, especially HNRNPF, PPP1R3G, IGF2BP1, TLR2, NT5E, BIRC3, PSCA, FGF2, MS4A1, CCT6A, ID1, TPM2 (Fig. 2k). As shown in Fig. 2l, as CDI increased, the survival rate of LUAD patients decreased.

The GSE68465 and GSE37745 cohorts were used to validate the predictivity of CDI signature. In the first step, we compared 1-, 2-, 3-, 4-, and 5-year AUC values of CDI signature with immunogenic cell death and necroptosis signatures using GSE68465 and GSE37745 (Supplementary Fig. 2a, h). The AUC values of 1–5 years of CDI signature were higher than those of immunogenic cell death and necroptosis signature. Kaplan–Meier survival curves were then used to examine the correlation between OS and CDI, immunogenic cell death, and necroptosis signature (Supplementary Fig. 2b–d, i–k). Compared with immunogenic cell death and necroptosis signature, there was a more significant prognostic difference between the high and low-risk groups stratified by CDI signature. Gene classification using UMAP was shown in GSE68465 and GSE37745 cohorts based on CDI (Supplementary Fig. 2e–g, l–n). Especially in Supplementary Fig. 2l, high and low-risk groups were significantly separated from each other. On the basis of the above findings, we concluded that CDI signatures are capable of predicting LUAD prognosis.

### The interaction of CDI signature with clinical variables

**Fig. 3:**
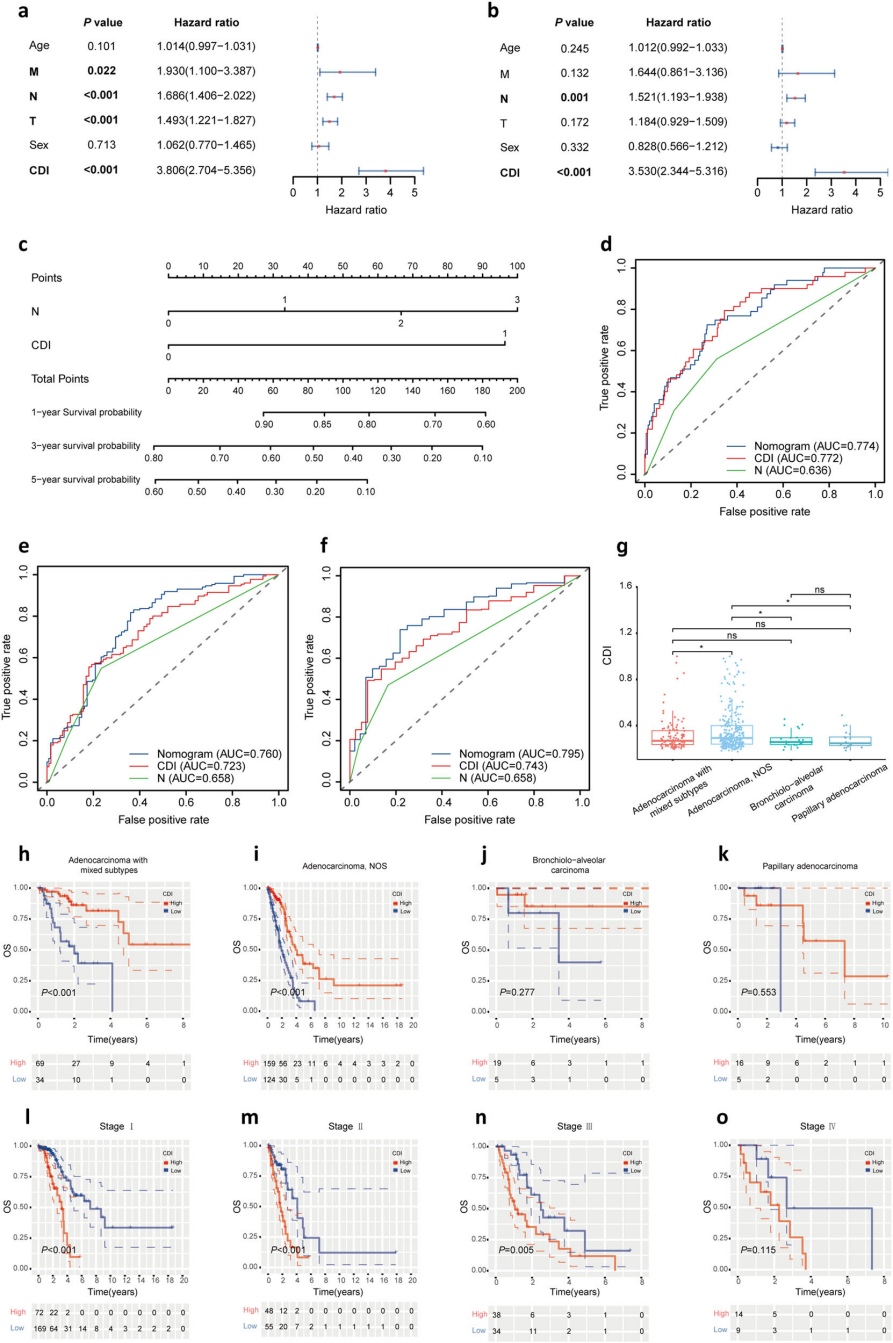
The interaction of CDI signature with clinical variables. **a**, **b** Univariate and multivariate Cox regression models for associations of risk score and clinicopathological factors with LUAD prognosis. **c** A nomogram consisting of CDI and N stage for predicting 1-, 3-, and 5- years survival for LUAD patients. **d–f** The 1-, 3-, and 5-year ROC curve of the nomogram, CDI, and N stage. **g** The CDI of LUAD histological phenotypes including adenocarcinoma with mixed subtypes, adenocarcinoma (NOS), bronchiolo-alveolar carcinoma, and papillary adenocarcinoma. **h–k** The K–M curve between high and low CDI group in adenocarcinoma with mixed subtypes, adenocarcinoma (NOS), bronchiolo-alveolar carcinoma, and papillary adenocarcinoma, respectively. **l**–**o** The K–M curve between high and low CDI group in LUAD with I, II, III, and IV stages. *P < 0.05, **P < 0.01, ***P < 0.001.

To investigate the interaction of CDI signature with clinical variables, we conducted univariate and multivariate Cox regression and concluded that CDI and N stage were independent prognostic factors for OS (Fig. 3a, b). By combining clinical factors including CDI and N stage, a nomogram was established as a novel prognostic model predicting the survival and guiding clinical decision-making in patients with LUAD (Fig. 3c). As shown in Fig. 3d, the 1-year AUC value of nomogram, CDI and N stage in the training set was 0.774, 0.772, and 0.636, respectively, showing the superior predictive performance of nomogram. We could get a similar conclusion from Fig. 3e, f.

As we all know, LUAD is a type of complex and highly heterogeneous disease with multiple histological phenotypes^23,24^. To predict better the prognosis of LUAD histological phenotypes, we first compared the CDI among several typical LUAD histological phenotypes including adenocarcinoma with mixed subtypes, adenocarcinoma (NOS), bronchiolo-alveolar carcinoma and papillary adenocarcinoma (Fig. 3g). It was shown that there was the significance of CDI between adenocarcinoma (NOS) and adenocarcinoma with mixed subtypes, bronchiolo-alveolar carcinoma as well as papillary adenocarcinoma. Furthermore, as for adenocarcinoma with mixed subtypes and adenocarcinoma (NOS) patients, the prognosis in the high CDI group was statistically worse than that in the low CDI group (*P* < 0.01) in Fig. 3h, i. However, we found no significance in high and low CDI in bronchiolo-alveolar carcinoma and papillary adenocarcinoma in Fig. 3j, k, further demonstrating the high heterogeneity of LUAD. We further explored the predictive value of CDI signature for the prognosis of patients with different TNM stages. As shown in the K–M curve of Fig. 3l–o, the survival of the low CDI group was significantly longer than that of the high CDI group in LUAD patients with I, II, and III stages. There was no significance between high and low CDI groups in LUAD patients with IV stage which might be due to the small number of IV stage LUAD patients enrolled.

### The comparison of CDI signature with other published prognosis biomarkers

**Fig. 4:**
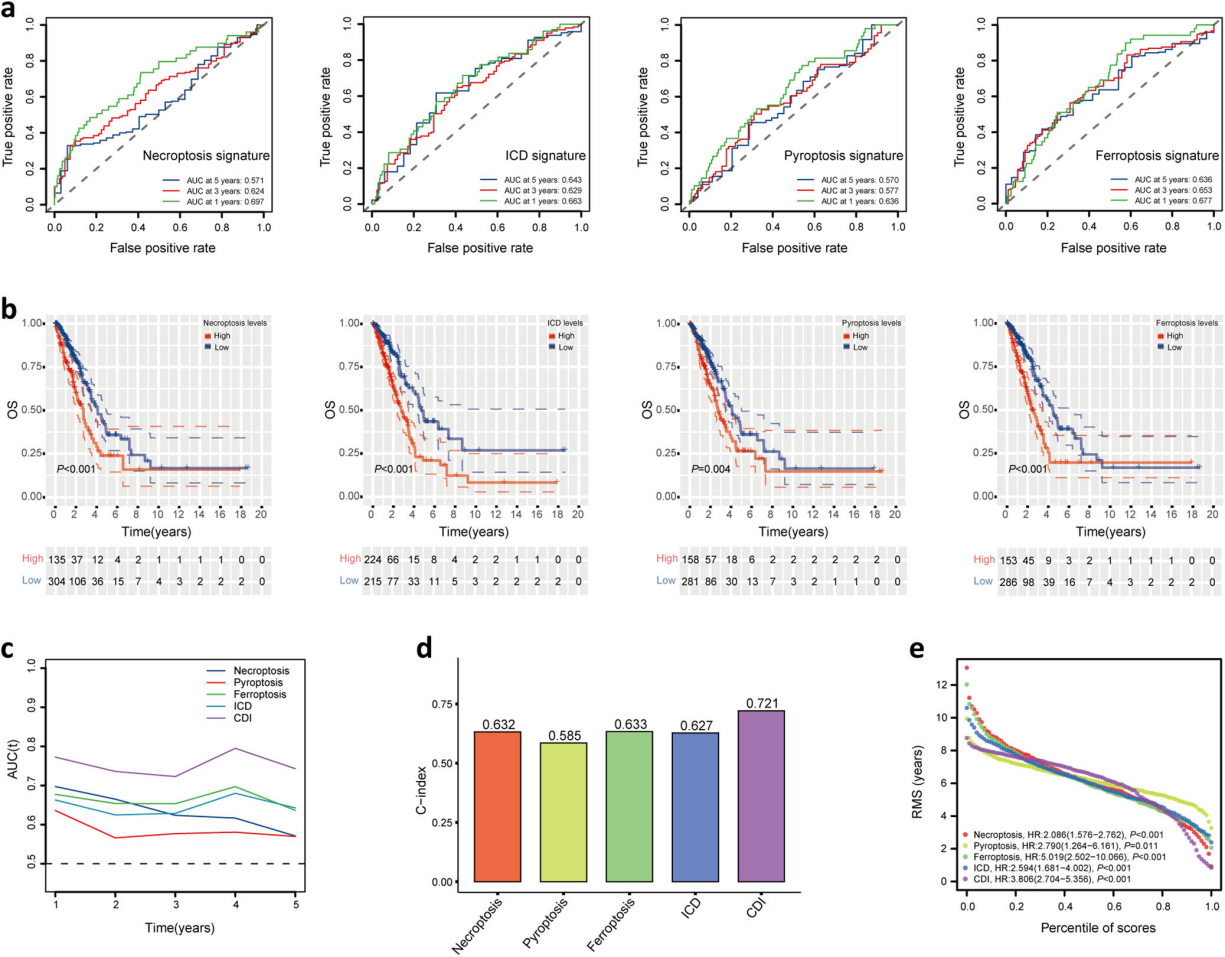
Comparison with other risk signatures. **a** ROC curve of Necroptosis signature, ICD signature, Pyroptosis signature, Ferroptosis signature. **b** K–M survival curve of the four signatures. **c** 1–5 years AUC values of CDI signature and four signatures. **d** C-index of our CDI signature compared with the four other signatures. **e** Restricted mean survival (RMS) curves for the five risk signatures.

A total of four signatures in previous studies including Necroptosis signature, ICD signature, Pyroptosis signature, and Ferroptosis signature were selected to compare with our CDI signature based on the TCGA LUAD cohort^25–28^. We calculated the risk score of each signature using the same methods to make these signatures comparable. The AUC at 1, 3, and 5 years of these four signatures were presented in Fig. 4a, respectively. Apparently, 1–5 years AUC values of our CDI signature were significantly higher than those of four published signatures (Fig. 4c). And compared with the four other signatures, the C-index of our CDI signature was the highest (Fig. 4d). As shown in K–M curve of the four signatures in Fig. 4b, the prognostic difference between high and low-risk groups was the most significant in our signature. Moreover, the hazard ratio and *p*-value of the five signatures were plotted in Fig. 4e. As a result, our CDI signature showed superior performance in prognosis prediction compared with the previous models.

### The association between immune cell infiltration and CDI signature

**Fig. 5:**
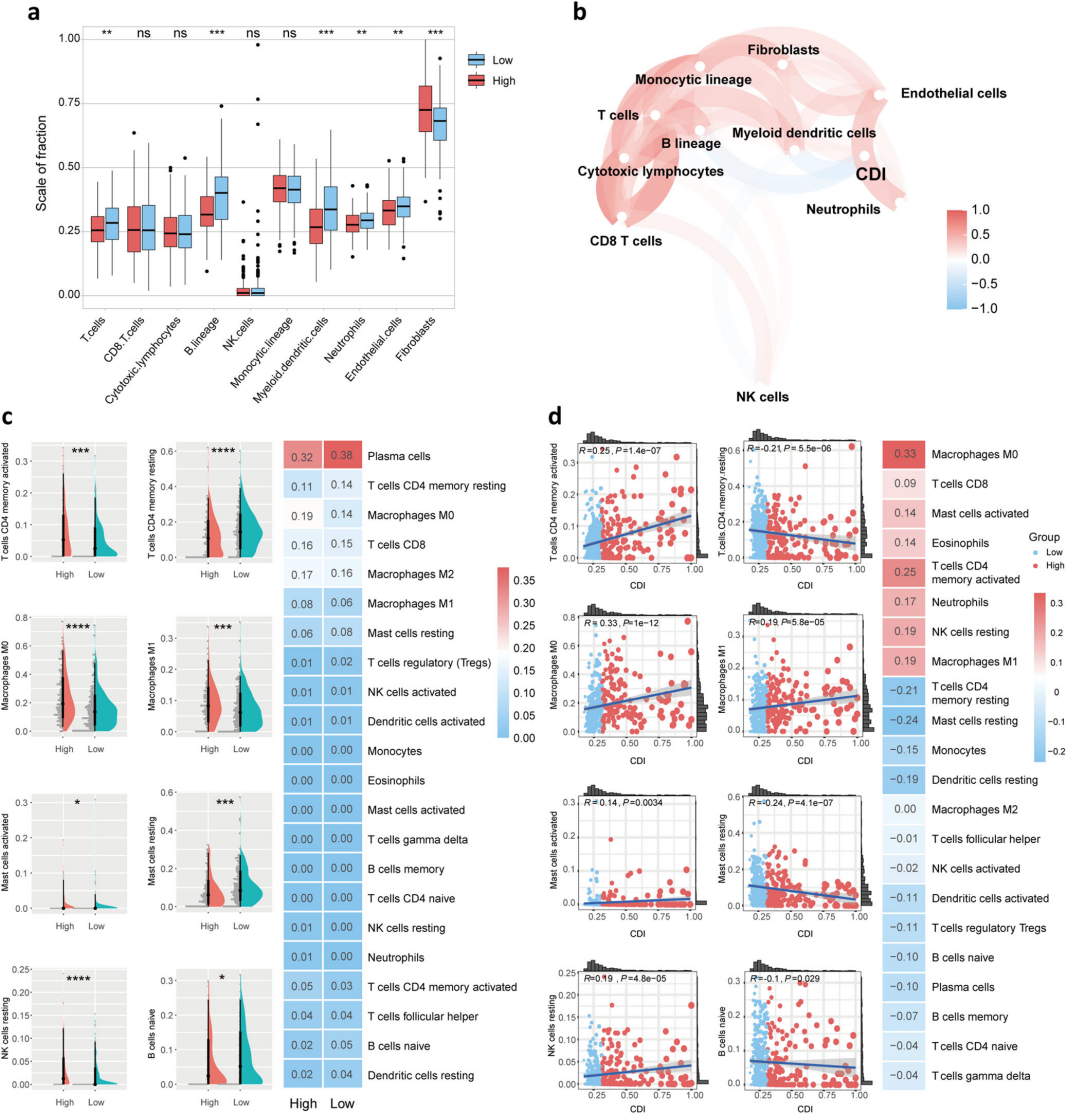
Correlation of CDI with immune cell infiltration. **a** The Wilcoxon rank-sum test compared the absolute abundance scores of 8 immune cells and 2 stromal cell populations in high and low CDI groups using the MCP counter. **b** The relationship of CDI with 8 immune cells and 2 stromal cell populations using MCP counter. **c** The difference of 22 immune cell infiltration levels between high and low CDI groups using the CIBERSORT algorithm. **d** The relationship of CDI with 22 immune cells using the CIBERSORT algorithm. *P < 0.05, **P < 0.01, ***P < 0.001, ****P < 0.0001.

A comparison of immune cell infiltration in low and high CDI groups was conducted to uncover the mechanism by which CDI is strongly associated with LUAD prognosis. MCP counter was used to compare the levels of infiltration of 8 immune cells and 2 stromal cells in high and low CDI groups (Fig. 5a). The absolute abundance score of T cells, B lineage, myeloid dendritic cells, neutrophils, and endothelial cells was statistically higher in low CDI group while that of fibroblasts was higher in high CDI group. CDI signatures were also correlated with immune cell populations and stromal cell populations using MCP counters in Fig. 5b. Additionally, we used the CIBERSORT algorithm to examine the difference between high and low CDI groups in terms of immune cell infiltration (Fig. 5c). The results showed that mast resting cells, naïve B cells, and CD4^+^ memory resting T cells were mainly enriched in low CDI group. In contrast, macrophage M0 and M1, CD4^+^ memory-activated T cells, and NK resting cells were significantly enriched in the high CDI group. In addition, the role of CDI signatures in 22 immune cell infiltration was explored using the CIBERSORT algorithm. The infiltration level of CD4^+^ memory-activated T cells, macrophage M0 and M1, mast-activated cells, and NK resting cells was positively associated with CDI while that of CD4 memory resting T cells, mast resting cells, and naïve B cells was negatively associated with CDI (Fig. 5d).

**Fig. 6:**
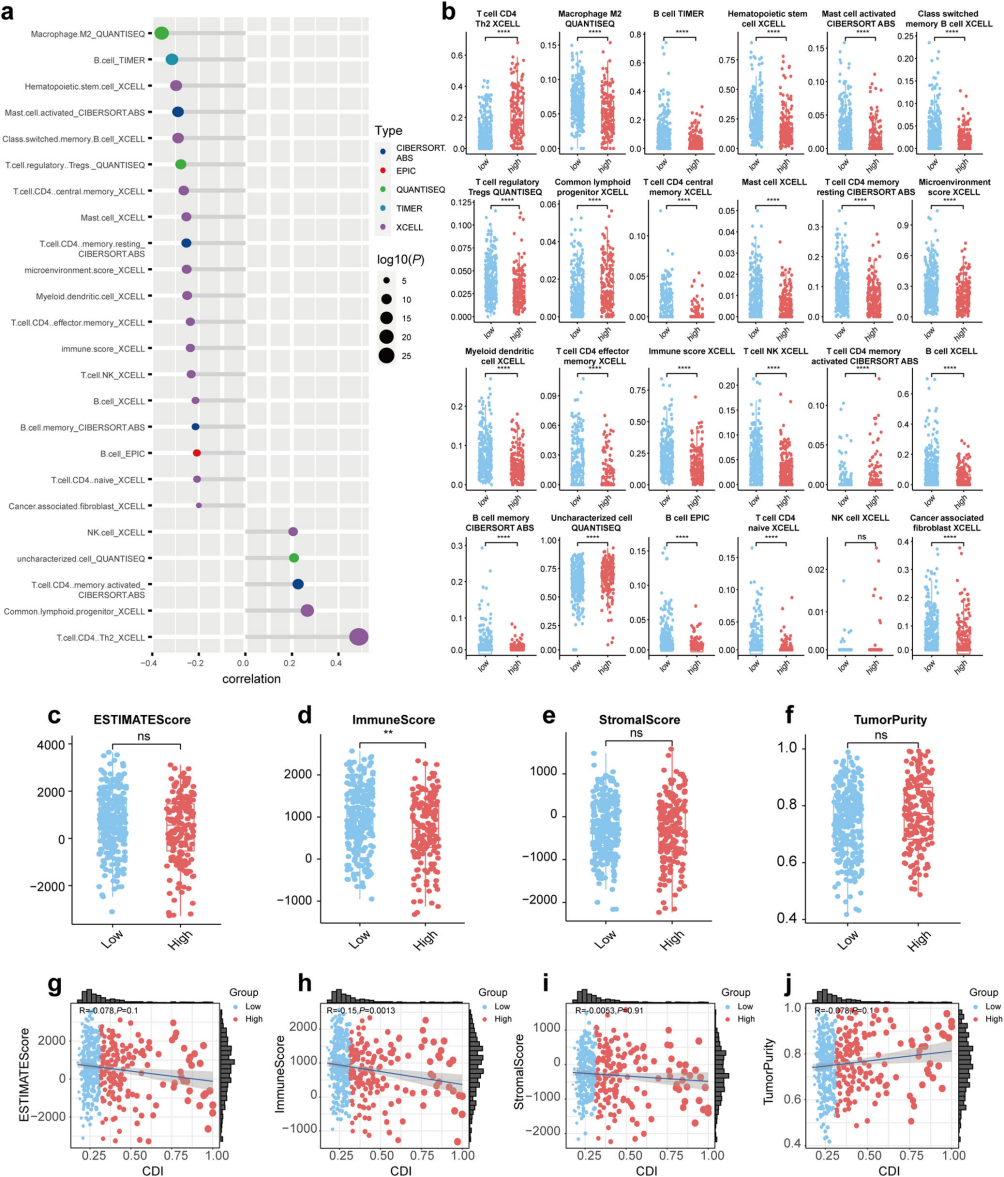
The investigation of the immune landscape in high and low CDI groups using several algorithms. **a** The correlation of CDI with infiltration level of immune cells using CIBERSORT, EPIC, QUANTISEQ, TIMER, and XCELL. **b** The difference in immune cell infiltration level between high and low CDI groups using CIBERSORT, EPIC, QUANTISEQ, TIMER, and XCELL. **c–f** The difference of ESTIMATE Score, IMMUNE Score, Stromal Score, and Tumor Purity between high and low CDI groups, respectively. **g–j** The relationship of CDI with ESTIMATE Score, IMMUNE Score, Stromal Score, and Tumor Purity, respectively. *P < 0.05, **P < 0.01, ***P < 0.001, ****P < 0.0001.

With several algorithms including CIBERSORT, EPIC, QUANTISEQ, TIMER, and XCELL, we provided insights into the immune landscape in low and high CDI groups to avoid inaccuracy and bias caused by the use of a single algorithm. As shown in Fig. 6a, CDI was correlated with the level of infiltration of immune cells based on CIBERSORT, EPIC, QUANTISEQ, TIMER, and XCELL. By using the algorithm described above, we were able to compare the levels of immune cell infiltration between high and low CDI groups in Fig. 6b. For example, the infiltration level of B cells was significantly higher in the low CDI group applying any of the abovementioned algorithms. We then applied the ESTIMATE algorithm to compare ESTIMATE Scores, Immunity Scores, Stromal Scores, and Tumor Purities between high and low CDI groups in Fig. 6c–f. IMMUNE Score was higher in the low CDI group and a significantly adverse correlation between IMMUNE Score and CDI was identified (*P* < 0.05) (Fig. 6g–j).

### Assessment of response to immunotherapy between high and low CDI groups

**Fig. 7:**
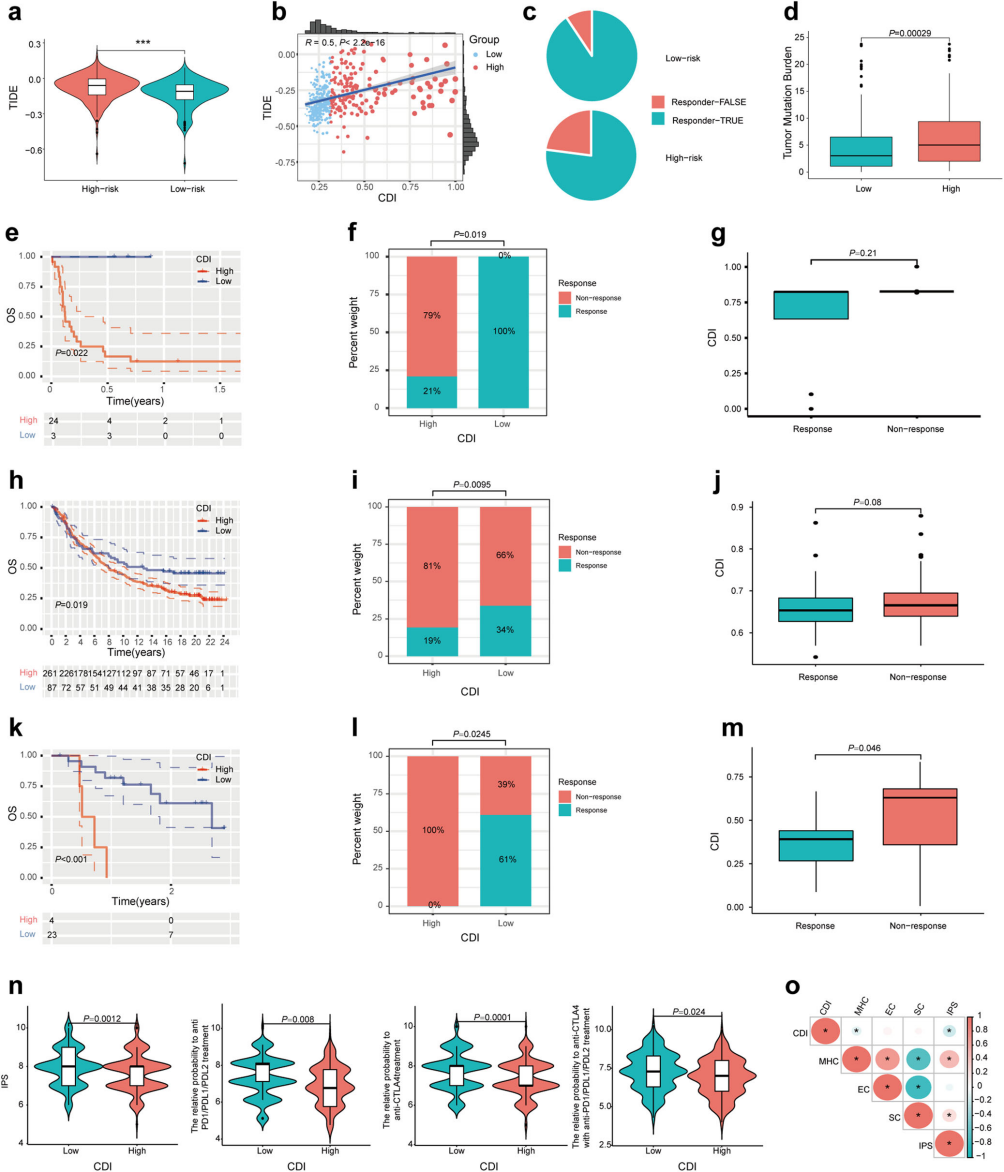
Correlation of CDI with immunotherapy-related biomarkers. **a** The difference in TIDE score between high and low CDI groups. **b** The relationship of CDI with TIDE score. **c** The percentage of immunotherapy responders and non-responders in high and low CDI groups using the TIDE score. **d** The difference of TMB between high and low CDI groups. **e**, **h**, **k** The Kaplan–Meier survival curve showed the relationship between OS and CDI in patients receiving immunotherapy with LUAD from GSE135222, metastatic urothelial carcinoma from IMvigor210 and melanoma from GSE78220, respectively. **f**, **i**, **l** The percentage of immunotherapy responders and non-responders in high and low CDI groups in patients receiving immunotherapy with LUAD from GSE135222, metastatic urothelial carcinoma from IMvigor210 and melanoma from GSE78220, respectively. **g**, **j**, **m** The difference of CDI between immunotherapy responders and non-responders in patients from GSE135222, IMvigor210, and GSE78220, respectively. **n** The difference of IPS between high and low CDI groups. **o** The relationship of CDI, MHC, EC, SC, and IPS in the TCGA cohort. *P < 0.05, **P < 0.01, ***P < 0.001.

In the modern era, cancer immunotherapy represented by ICIs represents a promising treatment option for recurrent and metastatic LUAD^29^. The TIDE score correlates closely with the immune escape from tumor microenvironments and resistance to immunotherapy. Higher TIDE scores indicate higher immune escape potential and lower immunotherapy response rates. According to Fig. 7a, b, TIDE scores in the low CDI group were significantly lower than those in the high CDI group, and CDI was positively correlated with TIDE scores (*R* = 0.5, *P* < 2.2e−16), demonstrating low CDI group had higher response rates of immunotherapy. Furthermore, we calculated the percentage of patients who responded to immunotherapy and those who did not respond using the TIDE score for the high and low CDI groups (Fig. 7c). As an increased TMB contributes to a stronger antitumor immune response^30^, we calculated TMB for each patient and examined the difference between high and low CDI groups in Fig. 7d. It was found that the TMB in the group with low CDI was lower than that of the group with high CDI. Following this, we examined the relationship between prognosis and CDI signature in the GSE135222 of patients with LUAD receiving immunotherapy^31,32^. According to the Kaplan–Meier survival curve, the overall survival of the low CDI group was higher than that of the high CDI group (*P* = 0.022) (Fig. 7e). In LUAD patients receiving immunotherapy, the proportion of responders and non-responders in high and low CDI groups was compared, as well as the CDI difference between immunotherapy responders and non-responders in Fig. 7f, g, respectively. The response rate was significantly higher in the low CDI group (*P* = 0.019) even though there was no significance of the CDI value between response patients and non-response patients (*P* = 0.21). Similar results were observed in patients receiving immunotherapy with metastatic urothelial carcinoma (MuC) from the iMvigor210 cohort and melanoma from GSE78220 (Fig. 7h–m). As presented in Fig. 7n, the relative probability to immunophenoscore (IPS) as well as immune checkpoint inhibitor including anti PD1/PDL1/PDL2, anti-CTLA4, and both treatments in the low CDI group was higher (*P* < 0.05). Moreover, we explored the correlation between CDI and immunotherapy-related biomarkers such as MHC molecules, effector cells (EC), immune checkpoints, and suppressor cells (SC). The anti-tumor immune response was promoted by MHC and EC while the immune response was suppressed by immune checkpoints and SC. The analogous result was also observed in Fig. 7o that the CDI was negatively linked with the expression of IPS and MHC while positively linked with the expression of EC and SC, although there was no significance.

## Gene set enrichment analysis and drug sensitivity of the CDI signature

Because of the intense relationship between the CDI signature and prognosis, immune microenvironment, and immunotherapy response of LUAD, we aim to apply gene set enrichment analysis to explore their biological process and internal connection. Using KEGG functional enrichment analysis, we found that the DEGs between high and low CDI populations were distinctively enriched in 9 KEGG pathways (*P* < 0.05). Among them, KEGG ECM RECEPTOR INTERACTION, CELL CYCLE, GAP JUNCTION, MISMATCH REPAIR, PATHWAYS IN CANCER, MAPK SIGNALLING PATHWAY, TGF BETA SIGNALLING PATHWAY, DNA REPLICATION were abundant in high CDI group and the low-CDI group had the most significant way in KEGG VEGF SIGNALLING PATHWAY (Supplementary Fig. 3a). To explore the relationship of tumor-related biological processes with CDI signature, gene set enrichment analysis (GSEA) was performed. As shown in Supplementary Fig. 3b, a total of 9 hallmark gene sets were enriched (*P* < 0.05). Hallmarks (including WNT BETA CATENIN SIGNALLING, EPITHELIAL MESENCHYMAL TRANSITION, G2M CHECKPOINT, E2F TARGETS, DNA REPAIR, CUPROPTOSIS, P13K AKT MTOR SIGNALLING, TNFA SIGNALLING VIA NFKB) were closely related with high CDI, indicating that the activation of these biological processes may play the vital role in tumorigenesis and progression. In contrast, the other hallmarks (PYROPTOSIS) were related to low CDI, implying that their activation participated in tumor suppression and prolonged prognosis in LUAD patients. We then estimated the IC50 value of drugs between high and low CDI groups in order to guide clinical treatment in LUAD. We found high CDI group tended to benefit from Paclitaxel, Gemcitabine, Vinblastine, Docetaxel, Cytarabine, Cisplatin, and Gefitinib (Supplementary Fig. 3c–j).

## High-resolution scRNA-seq revealed the immune landscape of LUAD

We analyzed 9 tumor tissue samples from 8 patients with LUAD to characterize the immune landscape in LUAD. All immune cells were classified into 10 cell types based on the expression level of canonical marker genes as reported previously using t-SNE method, including T cells, mono cells, B cells, epithelial cells, neutrophils, NK cells, mast cells, fibroblasts, endothelial cells and DC cells by well-recognized gene markers (Supplementary Fig. 4a). The UMAP plot has been uploaded in Supplementary Fig. 5. In the following step, we examined the fraction of different immune cell types in each cluster (Supplementary Fig. 4b). The results suggested that different immune cell types varied significantly among different clusters. To be specific, T cells were prevalent in C1–C6 and C9 while epithelial cells were predominant in C7 and C8. Moreover, the risk scores of necroptosis and ICD were calculated and demonstrated significantly different among different cell types in Supplementary Fig. 4c. Dotplot showed that most of the necroptosis and ICD-related model genes were generally expressed in each cell subtype and the expression value difference of the model genes in different cell subtypes further illustrated the heterogeneity within LUAD microenvironment (Supplementary Fig. 4d). Using t-SNE analysis, cancer-associated fibroblasts (CAF) were categorized as myCAF and iCAF cells. Development trajectory analyses of CAF cells further unveiled that the stage 1–2 and iCAFs were enriched in the initial differentiation phase while stage 5–7 and myCAFs were enriched in the terminal differentiation phase (Supplementary Fig. 4e–i). As shown in Supplementary Fig. 4j–l, the relationship of expression in BIRC3, CCT6A, HNRNPF, ID1, MYO6, and TPM2 with the pseudotime colored by state, cell subtype, and pseudotime, respectively was investigated.

## Cell communication network analysis in LUAD

To illustrate how immune cells regulate tumorigenesis, we studied the signaling pathways that allow multiple immune cells to interact with each other during tumorigenesis We described the number as well as weight and strength of interaction between immune cell types in Supplementary Fig. 6a, b. There was the largest number of interactions in epithelial–endothelial cells, mono–endothelial cells, mono cells–CAFs as well as the largest weight and strength of interaction in neutrophil–endothelial cells, mono cells–neutrophils, mono–endothelial cells. As shown in Supplementary Fig. 6c, d, the interaction number and weights/strength of CAF cells with other immune cell types were deeply explored. Furthermore, the role of related signaling pathways in cell–cell interaction was in-depth studied. According to our findings, immune cell interactions are closely related to three pathways involved in executive function (Supplementary Fig. 6e–g). The results showed that CAFs can receive the PERIOSTIN signaling not only sent by endothelial cells but also by CAFs themselves in the autocrine form. CAFs as signaling senders can transmit the activated MK signaling to multiple types of immune cells including epithelial cells, endothelial cells, DC cells, mono cells, T cells, B cells, NK cells, and mast cells. Similarly, ANGPTL signaling was also transmitted by CAFs to endothelial cells, neutrophils, and mono cells. Supplementary Fig. 6h, i illustrates the contribution weight of outgoing and incoming signaling patterns to immune cell types.

## Biological functions of the selected gene

**Fig. 8:**
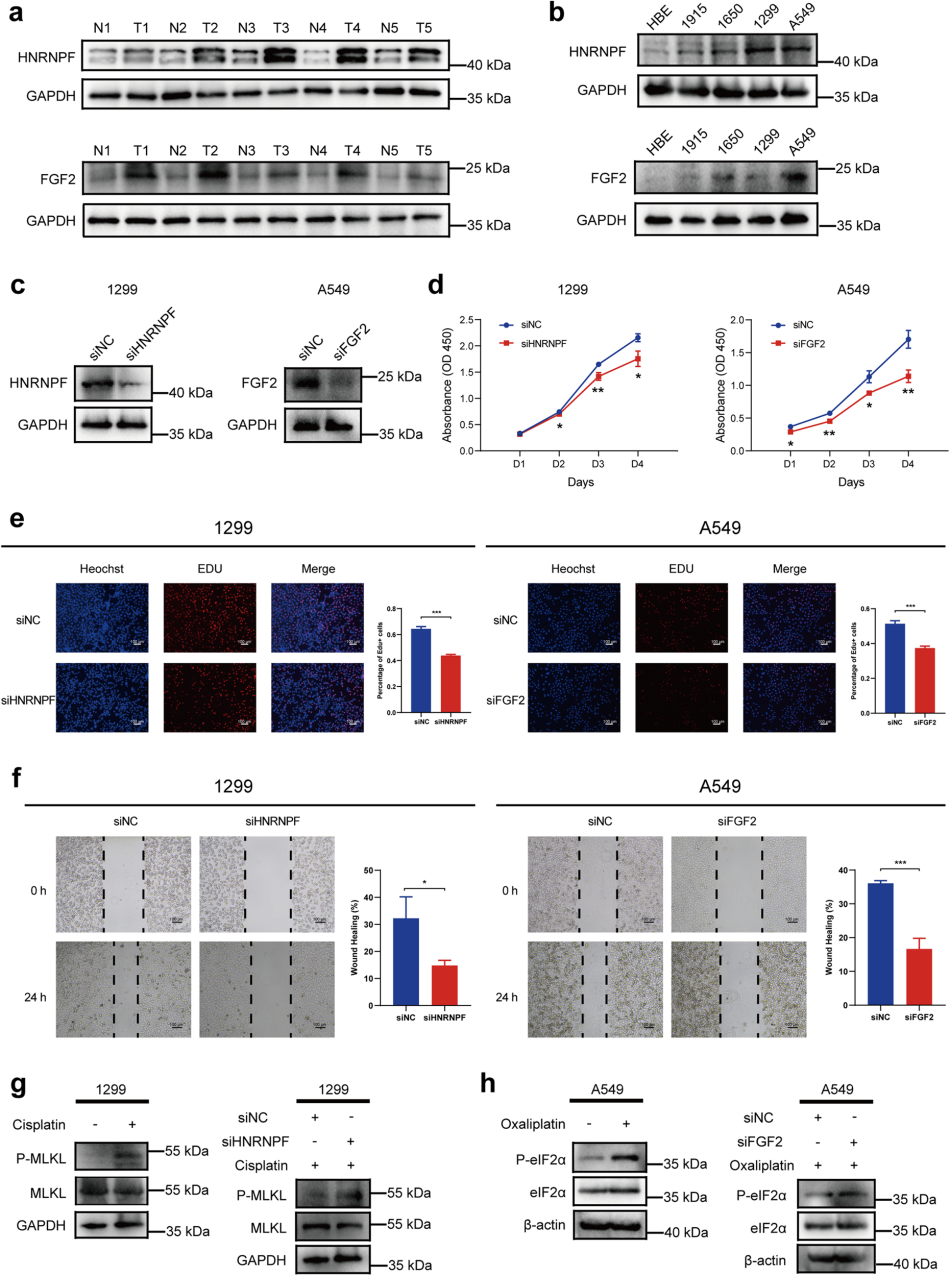
Validation of the potential function of HNRNPF and FGF2 in tumors by in vitro assays. **a** HNRNPF and FGF2 expression in five paired tumor tissues (T) and their adjacent normal tissues (N). **b** Comparison of HNRNPF and FGF2 expressions in human bronchial epithelial and lung cancer cells. **c** The expression of HNRNPF and FGF2 in 1299 and A549 cells, respectively, after transferring with siRNA. **d–f** Knockdown of HNRNPF and FGF2 inhibited the proliferation and migration of lung cancer cells by CCK-8 assay **d**, EDU assay **e**, and wound-healing assay (**f**). **g** The effects of cisplatin treatments on necroptosis in the 1299 cells were determined, and cisplatin-induced necroptosis was further increased in the HNRNPF silencing 1299 cells. **h** The effects of oxaliplatin treatments on ICD in the A549 cells were determined, and oxaliplatin-induced ICD was further enhanced in the FGF2-silencing A549 cells. *P < 0.05, **P < 0.01, ***P < 0.001.

To further verify the performance of CDI signature, we selected the HNRNPF and FGF2 that contributed the most to necroptosis and immunogenic cell death-related risk models, respectively. We first examined the expression of HNRNPF and FGF2 in five paired clinical LUAD specimens. The results showed that the expression of HNRNPF and FGF2 was elevated in the majority of tumor tissues (T) compared with the matched adjacent non-tumor tissues (N) (Fig. 8a). We next determined the expression levels of HNRNPF and FGF2 in HBE and different lung cancer cell lines (Fig. 8b). In the follow-up study, we conducted a knockdown analysis on H1299 cells with high expression of HNRNPF and A549 cells with high expression of FGF2 (Fig. 8c). The results of the CCK-8 assay and EDU assay proved that the knockdown of HNRNPF and FGF2 obviously inhibited lung cancer cells proliferation (Fig. 8d, e). Besides, the wound-healing assay demonstrated that the migration of cells was suppressed after the knockdown of HNRNPF and FGF2 (Fig. 8f).

Moreover, chemotherapy often elicits various cell death. Previous studies unveiled that cisplatin can induce necroptosis^33,34^. Based on the IC_50_ value of cisplatin in H1299 cells from the published literature^35^, we selected 10 μmol/L cisplatin for the validation of necroptosis. We found that the phosphorylation level of MLKL was upregulated following cisplatin treatment. The results suggested cisplatin stimulated necroptosis in H1299 cells, and knockdown of HNRNPF further increased cisplatin-induced necroptosis (Fig. 8g). Oxaliplatin is a known ICD inducer. Based on the IC_50_ value of oxaliplatin in A549 cells from the previous studies^36^, we selected 3 μmol/L oxaliplatin for the validation of ICD. Oxaliplatin promoted ICD-related proteins (P-eIF2α) expression in A549 cells, and knockdown of FGF2 further enhanced oxaliplatin-induced ICD (Fig. 8h).

## Discussion

The present study is the first comprehensive analysis of 15 types of cell death patterns, integrating necroptosis and ICD into CDI signature and validating its predictivity in TCGA and GEO cohorts. The CDI signature using necroptosis + immunologic cell death-related genes was established in the TCGA cohort with the 1-, 2-, 3-, 4-, and 5-year AUC values were 0.772, 0.736, 0.723, 0.795, and 0.743, respectively. The predictive performance was also verified in GSE37745 and GSE68465 successfully. We also investigated the relationship between the CDI signature and clinical variables, published prognosis biomarkers, immune cell infiltration, functional enrichment pathways as well as immunity biomarkers.

For decades, cell death has been implicated in biological processes underlying malignant tumor development and metastasis. Among cell death patterns, necroptosis was being increasingly investigated for its role in tumor formation, especially immune regulation^37^. The evidence suggested that tumor-specific antigens were provided to DCs by necrotrophic apoptotic cells, which then triggered cytotoxic CD8^+^ T cells^38^. RIPK3 regulated cytokine expression in DCs and might be involved in innate and adaptive immunity. Besides, a series of serine proteases is also involved in the necrotizing death pathway of neutrophils mediated by RIPK3-MLKL^39^. As immune cell recruitment to the tumor microenvironment is a promising therapeutic strategy, even in aggressive tumors, ICD has also gained increasing attention in the clinical management of cancer. For example, ICD played a crucial role in the response of colorectal adenocarcinoma cells to oxaliplatin in mice. This is because conventional chemotherapeutics exert tumor-suppressive effects mainly by inducing the release of DMAPs from cancer cells, activating the presentation of DC cells, and thus activating CD8^+^ T cells to kill cancer cells. The efficacy of these chemo agents varied in their ability to induce ICD.

Due to the strong correlation of necroptosis and ICD with immune regulation in tumor management, we applied several algorithms to explore the underlying relationship of CDI signature with immune infiltration. The findings showed that multiple types of immune cells including T cells, B cells, myeloid dendritic cells, neutrophils, and mast cells were more active in the low CDI group while other types of cells including cancer-associated fibroblasts (CAF), CD4^+^ memory-activated T cells, and NK resting cells were significantly enriched in high CDI group. Based on a large number of scientific studies, tumor-infiltrating lymphocytes (TILs) including T cells, B cells, and NK cells have been implicated in improving patient prognosis and immunotherapy efficacy in LUAD^40^. A detailed spatial analysis by Lopez de Rodas et al. found that lung cancer patients infiltrated with an amount of CD8^+^ T cells had a longer survival rate^41^. Based on WSI analysis of TILs, Park S., et al. also demonstrated higher immune cytolytic activities, higher response rates, and better prognosis in the immune phenotype characterized by high TIL density^42^. Aside from TILs, other immune cells within TME play a significant role in the progression and development of LUAD. For instance, a recent phase II study of the novel dendritic cell vaccine DCVAC/LuCa combined with standard carboplatin/pemetrexed for advanced LUAD showed promising results: the 1-year and 2-year survival rate was 72.73% and 52.57%, respectively^43^. Additionally, some inflammation-related pathways such as TGF BETA SIGNALLING PATHWAY and TNFA SIGNALLING VIA NFKB were identified to be associated with CDI signature as shown in GSEA analysis. In accordance with previous research and our findings, we further identified that CDI signature might be involved in the development of LUAD by regulating tumor immunity within TME.

With the advance of scRNA-seq, researchers have been able to identify CAF subpopulations and better understand CAF heterogeneity in a wide variety of tumor types. In our study, CAFs were categorized as myofibroblastic CAFs (myCAFs) and inflammatory CAFs (iCAFs) through t-SNE analysis. Notably, iCAFs marked by TPM2 were enriched in the initial differentiation phase while myCAFs characterized by BIRC3, CCT6A, HNRNPF, and ID1 were enriched in the terminal differentiation phase during tumor progression. As cancer evolves, CAFs may change dynamically in their characteristics and interactions with other cell types. The transcriptional profiles of CAF subsets in breast cancer shifted from immunoregulation to wound healing as well as antigen presentation, showing the dynamics of CAF subclusters during cancer development^44^ Similarly, recent research by Davidson S., etc., found that the abundance of three CAF subpopulations including stromal 1, stromal 2 and stromal 3 changed throughout tumor progression and stromal 3 subpopulations dominated tumor growth at later stages^45^. Furthermore, we identified that PERIOSTIN, midkine (MK), and angiopoietin-like (ANGPTL) were key signaling molecules in the interaction of CAFs with other immune cells.

In cancer treatment, immunotherapy has revolutionized the situation of patients with unresectable cancers^46^. Effective biomarkers for predicting immunotherapy efficacy include PD-1, PD-L1, MSI, TMB, etc. In spite of this, the relationship between these biomarkers is complicated and it is still unclear whether combining them is better than using one marker alone^47,48^. As an increased TIDE score indicates a greater likelihood of immune escape and less effectiveness of ICI treatment, the TIDE score was applied by our study in high and low CDI groups. Our study found that low-risk patients with low TIDE scores may benefit more from ICI therapy than high-risk patients with high TIDE scores. Additionally, the immune rejection (Exclusion) and immune dysfunction (Dysfunction) scores differed significantly between the two groups, further demonstrating the predictivity of CDI signature. IPS, primarily developed from TCGA RNA-seq data, was designed to predict patient responses to immune checkpoint inhibitor treatments^49^. It is confirmed that low-risk populations respond more effectively to immunotherapy, as evidenced by the higher IPS, which was consistent with our findings. As a result, it seems reasonable to assume that patients in the low CDI group benefit more from immunotherapy in terms of the treatment strategies for LUAD.

Tumor mutation burden (TMB) is referred to as the number of somatic mutations without germline mutations in the tumor genome. The high level of TMB was observed in the high CDI group, showing an increased tumor burden predicted the increasingly poor prognosis although higher TMB may contribute to a better immunotherapy response^50^. Multiple evidence in various tumor types has illustrated a relationship between the high level of TMB and the benefit from ICI which could be explained that there tends to be a higher amount of tumor neoantigens and a higher possibility of stimulating a stronger immune response in the tumor types with a higher TMB^51,52^. However, it has to be noted that TMB varies greatly across different tumor types in the TCGA database and kidney Chromophobe (KICH), diffuse large B-cell lymphoma (DLBC), adenoid cystic carcinoma (ACC), and low-grade glioma (LGG) had a better prognosis in the low TMB group. Thus, when exploring the impact of high TMB on tumor prognosis, the changes of OS in the high TMB group before and after ICIs should be considered. In addition to immunotherapy, the effect of TMB on molecular targeted therapies in LUAD was also explored. A recent study reported an inverse association between TMB and clinical response of EGFR tyrosine kinase inhibitors in patients with EGFR-mutant LUAD^53^. The scientific rationale might be that a high level of TMB can cause a high probability of resistance pathways as TP53 mutations can be significantly observed in patients with high TMB and TMB when progressing with EGFR-TKI is higher than pre-treating. We speculated that LUAD samples with driver genes mutation we downloaded from TCGA and GEO database are likely to receive TKI treatment, thus explaining why a high level of TMB was observed in the high CDI group. In general, TMB is an intriguing biomarker as its variable and therapy context-specific impacts on LUAD prognosis need to be deeply validated in routine clinical application.

In our study, we constructed a signature consisting of 14 CDI-related genes which was a promising predictor of LUAD prognosis including HNRNPF, PPP1R3G, IGF2BP1, TLR2, NT5E, BIRC3, PSCA, FGF2, MS4A1, CCT6A, ID1, TPM2, MYO6, H2AX. Among these genes, the genes that contributed the most to necroptosis and ICD-related risk models including necroptosis-related gene “HNRNPF” and ICD-related gene “FGF2”, respectively, were selected and performed in vitro experiments to verify the function of lung cancer. The knockdown of HNRNPF and FGF2 inhibited the proliferation and migration of lung cancer cells. We next determined that HNRNPF and FGF2 were involved in the cisplatin-induced necroptosis and oxaliplatin-induced ICD, respectively. It was observed that fibroblast growth factor 2 (FGF2) can activate FGFR1 to stimulate the proliferation, epithelial–mesenchymal transition (EMT), migration, and invasion in FGFR1-amplified lung cancer cell lines^54^. Moreover, FGF2 functioned as an angiogenic factor independent of VEGF in lung cancer cells determined by tube formation and neutralization assays^55^. RBM-007, an inhibitory RNA aptamer against FGF2, has been confirmed to have therapeutic effects on lung cancer in preclinical trials^56^. As an alternative splicing factor, HNRNPF played a major role in the inclusion and exclusion of cryptic exons^57^. It was reported to regulate alternative splicing in several cancer-associated processes, including epithelial to mesenchymal transitions and therapy resistance^58^. In thyroid cancer, alternative splicing mediated by HNRNPF can contribute to conventional cancer-related pathways including RTK/RAS/MAPK and PI3K/AKT/MTOR signaling^59^. By binding to the 3’ UTR of Snail1 mRNA, HNRNPF regulates EMT in bladder cancer^60^. Nevertheless, the regulatory mechanism of this gene in lung cancer is still poorly understood, which deserves further exploration in the future.

Although we conducted a comprehensive data analysis and multiple data validations in our study, some limitations and shortcomings remain. Firstly, there were a limited number of patients and all data were obtained from public databases. Therefore, more clinical data are needed to validate this prognostic model. Secondly, clinicopathological information from TCGA and GEO databases was incomplete so the CDI might not be an independent predictor of LUAD prognosis. Lastly, the mechanism of CDI-related genes in LUAD prognosis remains unknown, a more in-depth investigation of these genes in LUAD development will be undertaken in vivo or in vitro.

In conclusion, the CDI signature established in this study is a novel prognostic predictor that uncovers new immunotherapy targets and new theoretical foundations for LUAD diagnosis, prognosis assessment, and individual treatment.

## Methods

### Data collection

We selected the key regulatory genes for 15 cell death patterns including pyroptosis, ferroptosis, necroptosis, autophagy, immunologic cell death, entotic cell death genes, cuproptosis, parthanatos, lysosome-dependent cell death, intrinsic apoptosis, extrinsic apoptosis, necrosis, anoikis, apoptosis-like morphology, and necrosis-like morphology using a combination of gene set enrichment analyses (GSEA) gene sets from MSigDB (http://software.broadinstitute.org/gsea/msigdb/index.jsp), Kyoto Encyclopedia of Genes and Genomes (KEGG), review articles, and manual collection of gene sets from Genecards website (https://www.genecards.org/)^61,62^. GeneCards is an integrative human gene database in which there is highly comprehensive and user-friendly gene information to help us better investigate human genetic research (Supplementary Table 1).

Raw bulk transcriptome counts, normalized and log2 converted RNA-sequencing profiles FPKM as well as clinical information for LUAD patients and normal samples were identified from the the Cancer Genome Atlas (TCGA) database (https://portal.gdc.cancer.gov/). The IMvigor210 dataset from http://researchpub.gene.com/IMvigor210CoreBiologies. We downloaded the bulk RNA-seq datasets (GSE37745, GSE68465, GSE135222, and GSE78220) and the single-cell RNA-seq dataset (GSE171145) from the Gene Expression Omnibus (GEO) database (http://www.ncbi.nlm.nih.gov/geo/). Among them, GSE171145 (including 40,799 single cells from 9 LUAD samples for LUAD patients) was analyzed by “Seurat V4” R package.

### Identification of cell death-related genes associated with LUAD prognosis

Clinical data including overall survival (OS) were identified from the TCGA database. We excluded the samples without survival-related information. First, prognosis-associated genes in 15 cell death types were screened using univariate Cox regression. Then, the cell death-related genes tightly linked to LUAD prognosis were further screened using least absolute shrinkage and selection operator (LASSO) analysis which was incorporated into the multivariate Cox regression model. Furthermore, we plotted the forest plots of selected genes using the R package “forestplot” through multivariate Cox analysis to explore the independent predictor of OS. Before establishing the risk score model, the residual method was used to test the equal proportional risk hypothesis of the studied variables. The principle of the residual method test is that the residual does not change significantly with the change of time (*P* > 0.05), indicating that the variable conforms to the assumption of equal proportional risk. Then we calculated the risk score model based on the model formula: Risk score = ΣiCoefficient (mRNAi) × Expression (mRNAi), divided into high and low-risk groups according to the best cut-off values of risk score and then compared the different expressions of the screened genes in the high and low-risk groups.

### The establishment and validation of cell death-related prognosis signature

Based on the following model formula: CDI = ΣiCoefficient (mRNAi) × Expression (mRNAi), we calculated the CDI of each patient using prognostic cell death-related gene expression. The cut-off value was determined by the “surv_cutpoint” function of the R package “survminer”, which calculates statistics based on maximally selected rank statistics. The methods to determine the best cut-off value of the function include the minimum *P*-value method and the maximum statistics method. The minimum *P*-value method refers to calculating the *P*-value between high and low-risk groups according to the different cut-off values until the *P*-value is the minimum. The maximum statistic method refers to calculating the statistics of the log-rank test between high and low-risk groups according to different cut-off values until the statistic is maximum. Besides, we standardized the included data in this study, and the criteria for logarithmic conversion of the raw data was consistent, so the same cut-off values were selected across all cohorts. Through time-related receiver operating characteristic (ROC), the predictive reliability of the cell death-related signature was assessed. To identify the combined cell death-related signatures with the highest AUC values, we applied an exhaustive search algorithm in the R package “leaps” to assess a series of candidate generalized linear models (GLMs) that contained different combinations of cell death types^63^. Among 15 types of cell death, we selected a cell death-related risk score model with AUCs >0.7, paired them, and finally got 28 cell death combination types (Supplementary Table 2). Based on the AUC values, the best CDI signature was chosen and then applied to predict the prognosis of LUAD patients. The Kaplan–Meier survival curve was performed to compare the OS between the high and low CDI groups using R packages “survival” and “survminer”.

### Construction of the nomogram

Nomogram is a powerful tool in tumor prognosis prediction, which integrates multiple clinicopathology prognostic variables and calculates the occurrence possibility of individual clinical events^64^. The nomogram predicting 1-, 3-, and 5-year OS of LUAD patients was established based on the CDI signature and clinicopathology factors through the “rms” R package. Besides, the ROC curve was applied to evaluate the accuracy of the nomogram in predicting LUAD prognosis.

### Immune infiltration and biomarkers analysis

The TIMER2.0 database was applied to explore the relationship between CDI signature and immune infiltration was identified (http://timer.comp-genomics.org). Immunedeconv, an R package integrating six state-of-the-art algorithms including TIMER, xCell, MCP-counter, CIBERSORT, EPIC, and quanTIseq was utilized^65^. Each algorithm was systematically benchmarked and found to have unique properties and strengths. The MCP-counter generates absolute abundance scores for ten immune cell and stromal cell populations based on the normalized FPKM expression matrix converted by log2^66^. Besides, the enrichment of 22 immune cells was inferred by the CIBERSORT algorithm^67^. CIBERSORT can compute the abundance of specific cell types in a mixed sample based on the bulk expression. The ESTIMATE (Estimation of Stromal and Immune cells in Malignant Tumor tissues using Expression data) algorithm was also used to analyze the difference of stromal score, immune score, and ESTIMATE score by the R package “estimate”^68^.

Two primary mechanisms of tumor immune evasion were modeled using the algorithm tumor immune dysfunction and exclusion (TIDE). We uploaded the processed expression profile matrix of LUAD patients to the TIDE database online website (http://tide.dfci.harvard.edu/) to derive per patient’s TIDE score for predicting immunotherapy response. We also calculated the immunophenoscore (IPS) for LUAD patients from The Cancer Immunome Atlas (TCIA, https://tcia.at/home). The IPS is obtained according to four significant tumor immunogenicity-related aspects, including effector cells (EC), immunosuppressive cells (SC), major histocompatibility complex (MHC) molecules which are characterized by antigen processing as well as checkpoints/immunomodulators. We identified the IPS which ranged from 0 to 10 according to the *z*-score of related gene expression. Besides, the number of somatic non-synonymous mutations in the given genomic region is usually called tumor mutation burden (TMB) which is exhibited as enzyme mutations per Megabyte (mut/Mb). TMB is highly related to the neoantigen production within the tumor microenvironment and is thus used to predict the immunotherapy response in multiple tumor types. TMB data of LUAD patients was downloaded by “mutect2” algorithm using the R package “TCGAmutations”.

### Gene set enrichment analyses

Using the R packages “clustersProfiler”, “enrichplot”, and “ggplot2”, we performed gene set enrichment analyses (GSEA) to improve our understanding of CDI signature function and pathway. The gene sets “c2.cp.kegg.v7.4. symbols.gmt” and “h.all.v7.4.symbols.gmt” was chosen as the reference gene set. The normalized enrichment score (|NES | >1), nominal *P-*value < 0.05 (NOM *P-*value), and FDR adjusted *q*-value < 0.25 were considered as significant pathway enrichment^69–72^.

### Visualization

ScRNA-seq data were quality-controlled prior to analysis, and cells with >25% of mitochondria-associated genes were filtered out. The top 2000 highly variable genes of each sample were normalized using the ScaleData function based on variance stabilization transformation (vst). The dimensionality of the PCA was reduced using the RunPCA function. We chose dim = 20 and clustered the cells into different cell groups using “FindNeighbors” and “FindClusters” functions. The resolution was 0.5. t-distributed stochastic neighbor embedding (T-SNE) and uniform manifold approximation and projection (UMAP) nonlinear dimension reduction methods in seurat were applied, to map high dimensional cellular data into a two-dimensional space, grouping cells with similar expression patterns and separating those with different expression patterns. T-SNE can retain local structures between samples by optimizing the stochastic divergence of Kullback–Leibler, which has better advantages in visualizing high–low-risk groups. It can also test whether there are overlapping clustering and outlier samples^73^. UMAP, through error optimization, makes similar samples closer thus visualization effect is more stable, and more global structure information can be retained^74^. Both algorithms set the seed number to “123456789”. Two algorithms are used to visualize the results after NMF clustering, which can further verify the accuracy of the clustering results. As a result, the differences between cells became more apparent. In the following step, we made annotations for each cell type using SingleR. The reference cells were used by SingleR to identify cell types in order to identify the similar expression patterns between the cells.

### Pseudotime analysis

An analysis of pseudotime, also known as cell trajectory analysis, helps predict the evolutionary trajectory of apoptosis pathways and cell subtypes and infer the differentiation trajectory of stem cells during disease progression. By analyzing key gene expression patterns using Monocle 2, we performed pseudotime analysis in the current study. The pseudotime value was used by Monocle to model the gene expression level as a nonlinear smooth pseudotime function to show changes in gene expression with time. FDR <1e−5 was regarded as a significant difference.

### Cell–cell interaction analysis

Based on the ligand–receptor information, we used the single-cell gene expression matrix to unravel the communication between immune cell subtypes which was contained in CellChat software (http://www.cellchat.org/) with default parameters, modeling the communication probability and identifying significant communications.

### Cell culture and treatment

The Human bronchial epithelial cells (HBE) and a collection of lung cancer cells (A549, NCI-H1299, NCI-H1915, and H1650) were obtained from the Laboratory of Medical Genetics (Department of Biology, Harbin Medical University, Harbin, China). Cells were cultured in the DEME (HBE), or RPMI 1640 (A549, H1299, H1915, and H1650 cells) medium (Gibco, Invitrogen) supplemented with 10% fetal bovine serum (PAN-Biotech, Germany), penicillin G (100 U/ml, Beyotime, China) and streptomycin (100 μg/ml, Corning, China). Cell cultures were kept in a humidified incubator at 37 °C with 5% CO_2_. Cisplatin and oxaliplatin were obtained from Qilu Pharmaceutical (China) and dissolved in sterile water. The cells were treated with cisplatin and oxaliplatin for 72 h before the follow-up experiments.

### Western blot analysis

Total proteins from LUAD specimens and cells were extracted with RIPA (Beyotime, China) buffer supplemented with a phenylmethanesulfonyl fluoride (Beyotime, China) for 20 min. Equal amounts of proteins were electrophoresed in SDS–PAGE (10%) and transferred to PVDF membranes. After blocking with 5% skimmed milk in TBST at room temperature for 1 h, the membranes were incubated overnight at 4 °C with primary antibodies against HNRNPF (1:1000, Affinity), FGF2 (1:1000, Affinity), MLKL (1:1000, Affinity), P-MLKL (1:1000, Affinity), eIF2α (1:1000, Affinity), P-eIF2α (1:1000, Affinity), β-actin (1:1000, Beijing Zhongshan Golden Bridge Biotechnology Co.Ltd), and GAPDH (1:1000, Absin), as needed. After washing with TBST, the membranes were incubated with a secondary antibody (1:10,000, Beijing Zhongshan Golden Bridge Biotechnology Co. Ltd) for 1 h at room temperature. Finally, an ECL detection system (Beyotime, China) was used to detect targeted protein bands. GAPDH and β-actin were used as the internal controls.

### Patients and samples

We collected fresh tissue specimens from five patients with LUAD receiving surgical operations in the Harbin Medical University Cancer Hospital. These patients did not receive any anticancer treatments prior to surgery. The approval of this study was obtained from the Ethics Committee of Harbin Medical University Cancer Hospital. The study was conducted in accordance with the Declaration of Helsinki, and written informed consent was obtained from all patients prior to participation.

### Assay for proliferation and migration

The H1299 cells were transfected with HNRNPF siRNA (RiboBio, China) to knock down the HNRNPF expression according to the manufacturer’s protocols; the A549 cells were transfected with FGF2 siRNA (RiboBio, China) to knock down the FGF2 expression according to the manufacturer’s protocols. The sequences of HNRNPF-siRNA and FGF2-siRNA were designed according to a previous study^75,76^. The siRNA duplex sequences used to target HNRNPF (HNRNPF-siRNA) were as follows: sense, 5′-CCGCAGGUGUCCAUUUCAUTT-3′; and antisense, 5′-AUGAAAUGGACACCUGCGGTT-3′. The siRNA duplex sequences used to target FGF2 (FGF2-siRNA) were as follows: sense, 5′-GGAGUGUGUGCUAACCGUUTT-3′; and antisense, 5′-AACGGUUAGCACACACUCCTT-3′.

The proliferation of H1299 and A549 cells was assessed with the cell counting kit-8 (CCK-8) assay (APExBIO, USA). About 4 × 10^3^ cells were inoculated into 96-well plates, and cultured for 24, 48, 72, and 96 h. Subsequently, the OD values were measured at 450 nm with a microplate reader.

The 5-Ethynyl-20-Deoxyuridine (EDU) incorporation assay kit (RiboBio, China) was used to measure the proliferation capacity of cells. About 3 × 10^3^ cells were seeded into 96-well plates per well. After 48 h, the cells were incubated with a culture medium containing EDU for 2 h at 37 °C. Finally, A fluorescence microscope (Olympus, Japan) was utilized to capture images.

We also evaluated cell migration using the wound-healing assay. While cells reached confluence, we scratched the cell layer with the tip of a 1000 µL pipette tip. And afterward, a serum-free medium was used to maintain cells. The wounded areas were photographed under a light microscope (Nikon, Japan) when the wound was created (0 h) and 24 h later.

### Statistical analysis

R version 4.1.3 was used for all statistical studies. The survival curve was plotted by the Kaplan–Meier survival curve and the *P*-value was obtained by the log-rank test. The Student’s *t*-test and Wilcoxon test were used to compare the differences between the two groups, and Spearman analysis was used to calculate the correlation coefficients. Double-tailed *P* < 0.050 was considered statistically significant.

### Reporting summary

Further information on research design is available in the Nature Research Reporting Summary linked to this article.

### Supplementary information

Supplementary Information file

Supplementary Data 1

Supplementary Data 2

REPORTING SUMMARY

## Data Availability

The data supporting this study’s findings are available in the methods and/or supplementary material of this article. TCGA-LUAD was downloaded from https://portal.gdc.cancer.gov/. The IMvigor210 dataset from http://researchpub.gene.com/IMvigor210CoreBiologies. GSE37745, GSE68465, GSE135222, GSE78220, and GSE171145 are available in the GEO (https://www.ncbi.nlm.nih.gov/geo/) dataset. Further inquiries can be directed to the corresponding author.

## Code availability

The R codes (R version 4.1.3) for the statistical analyses and for figure generation are available from the corresponding author upon request.
