# Supplementary material for: Implications of different cell death patterns for prognosis and immunity in lung adenocarcinoma
Source: NPJ Precis Oncol. 2023 Nov 15;7:121. doi: 10.1038/s41698-023-00456-y (PMC10651893; doi:10.1038/s41698-023-00456-y)
Supplement: Supplementary file 1 — Supplementary Information file [file 41698_2023_456_MOESM1_ESM.pdf]

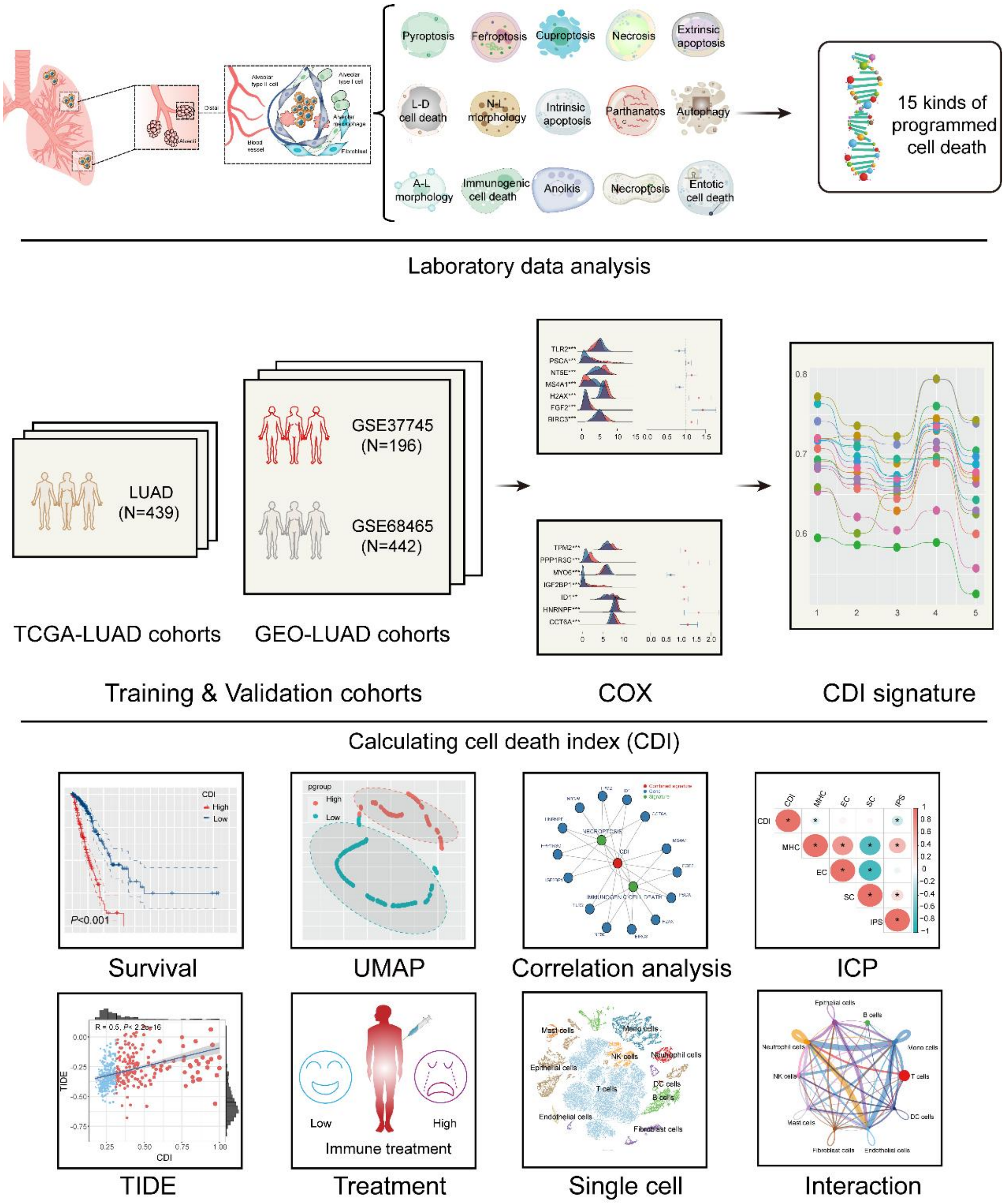

Supplementary Fig. 1 The flow chart of our study.

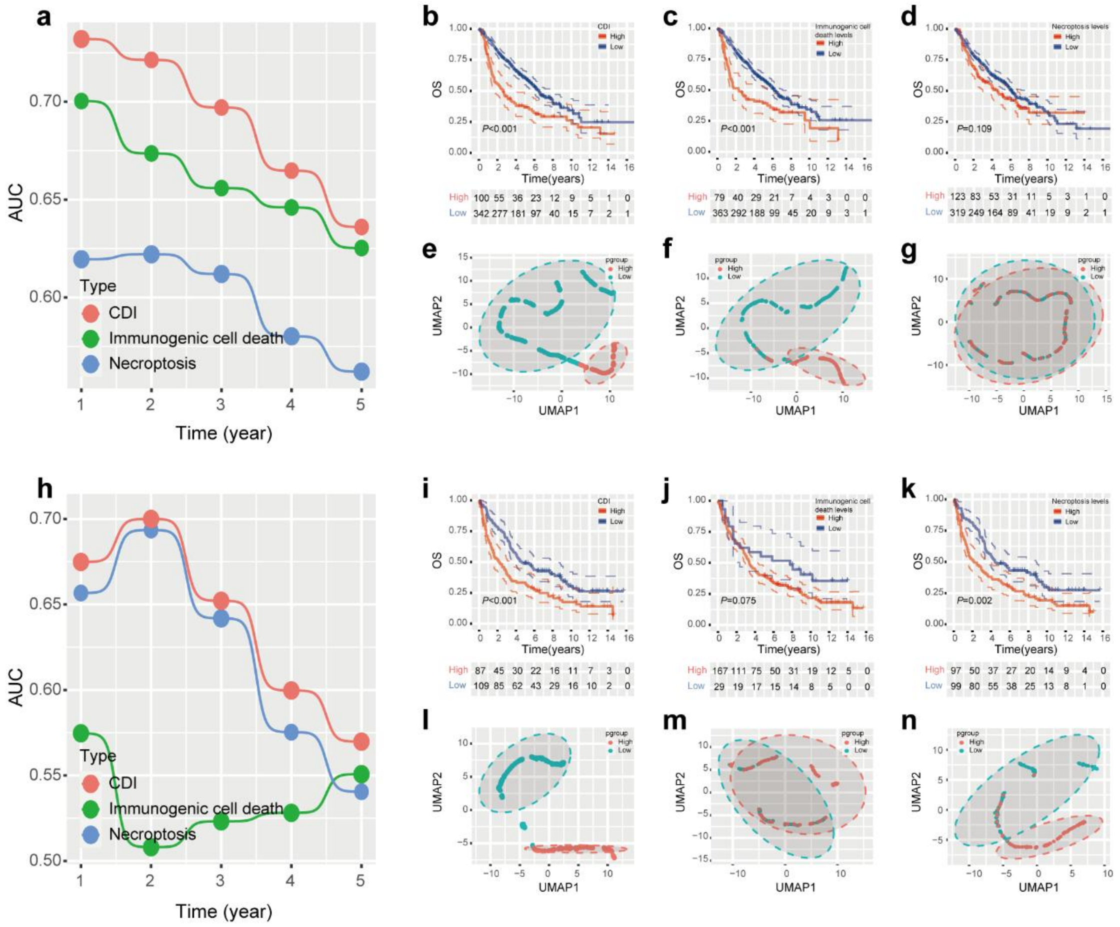

Supplementary Fig. 2 The validation of CDI signature in prognosis of LUAD. a A comparison of 1-,2-,3-,4- and 5-year AUC values of CDI signature with necroptosis and immunologic cell death signature using GSE68465 cohort. **b-d** The Kaplan-Meier survival curve with log-rank test using GSE68465 cohort demonstrated the relationship between OS and CDI signature, necroptosis and immunologic cell death signature, respectively. **e-g** Clustering analysis using GSE68465 cohort showed gene classification in high and low risk groups based on CDI signature, necroptosis and immunologic cell death signature, respectively. **h** A comparison of 1-,2-,3-,4- and 5-year AUC values of CDI signature with necroptosis and immunologic cell death signature using GSE37745 cohort. **i-k** The Kaplan-Meier survival curve with log-rank test using GSE37745 cohort demonstrated the relationship between OS and CDI signature, necroptosis and immunologic cell death signature, respectively. **l-n** Clustering analysis using GSE37745 cohort showed gene classification in high and low risk groups based on CDI signature, necroptosis and immunologic cell death signature, respectively.

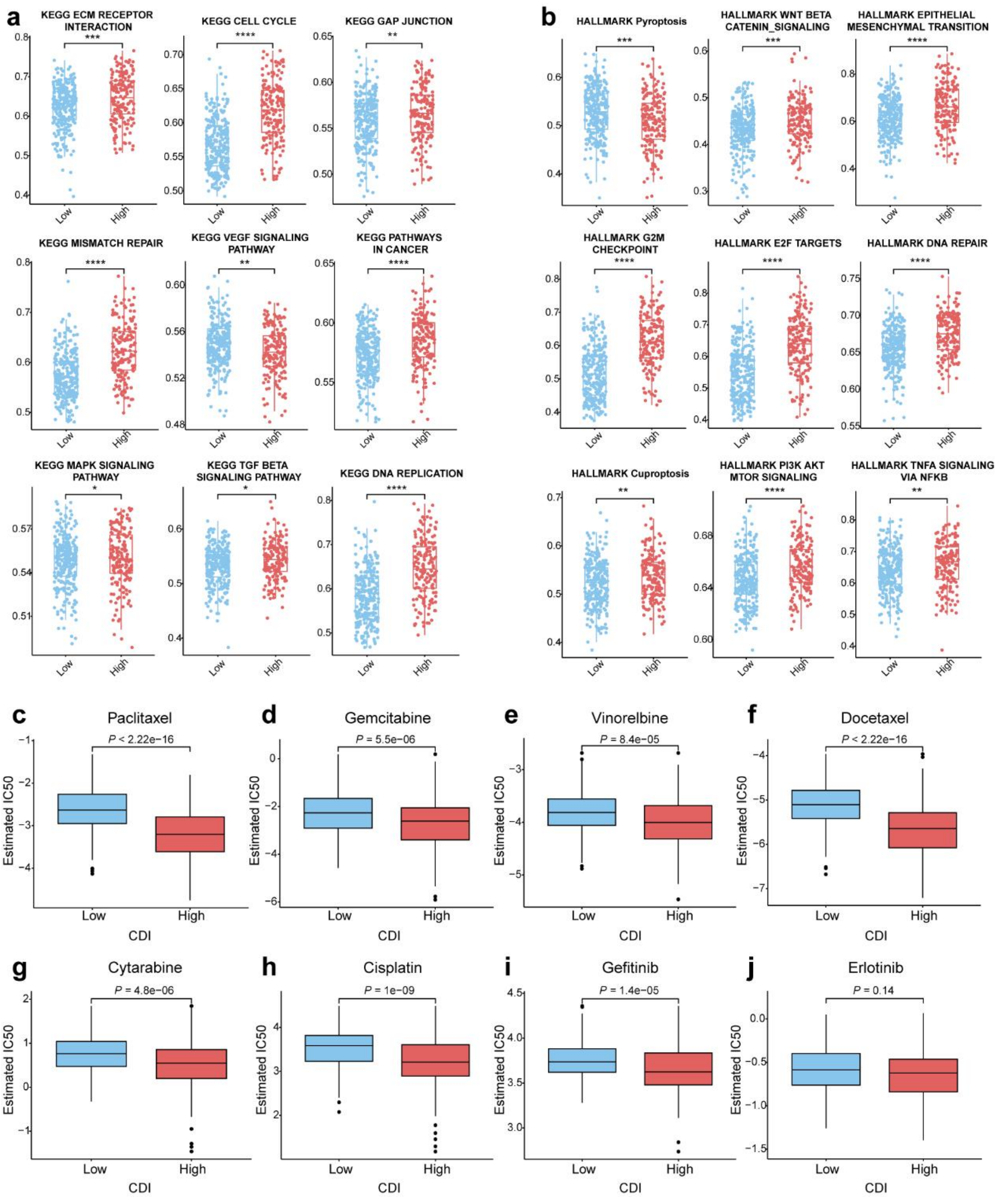

Supplementary Fig. 3 Gene set enrichment analysis and drug sensitivity of the CDI signature. **a** The significantly enriched subset of KEGG canonical pathways based on GSEA. **b** The significantly enriched HALLMARK gene sets based on GSEA. **c-j** Drug sensitivity of the CDI signature. **P*<0.05, ***P*<0.01, ****P*<0.001, *****P*<0.0001.

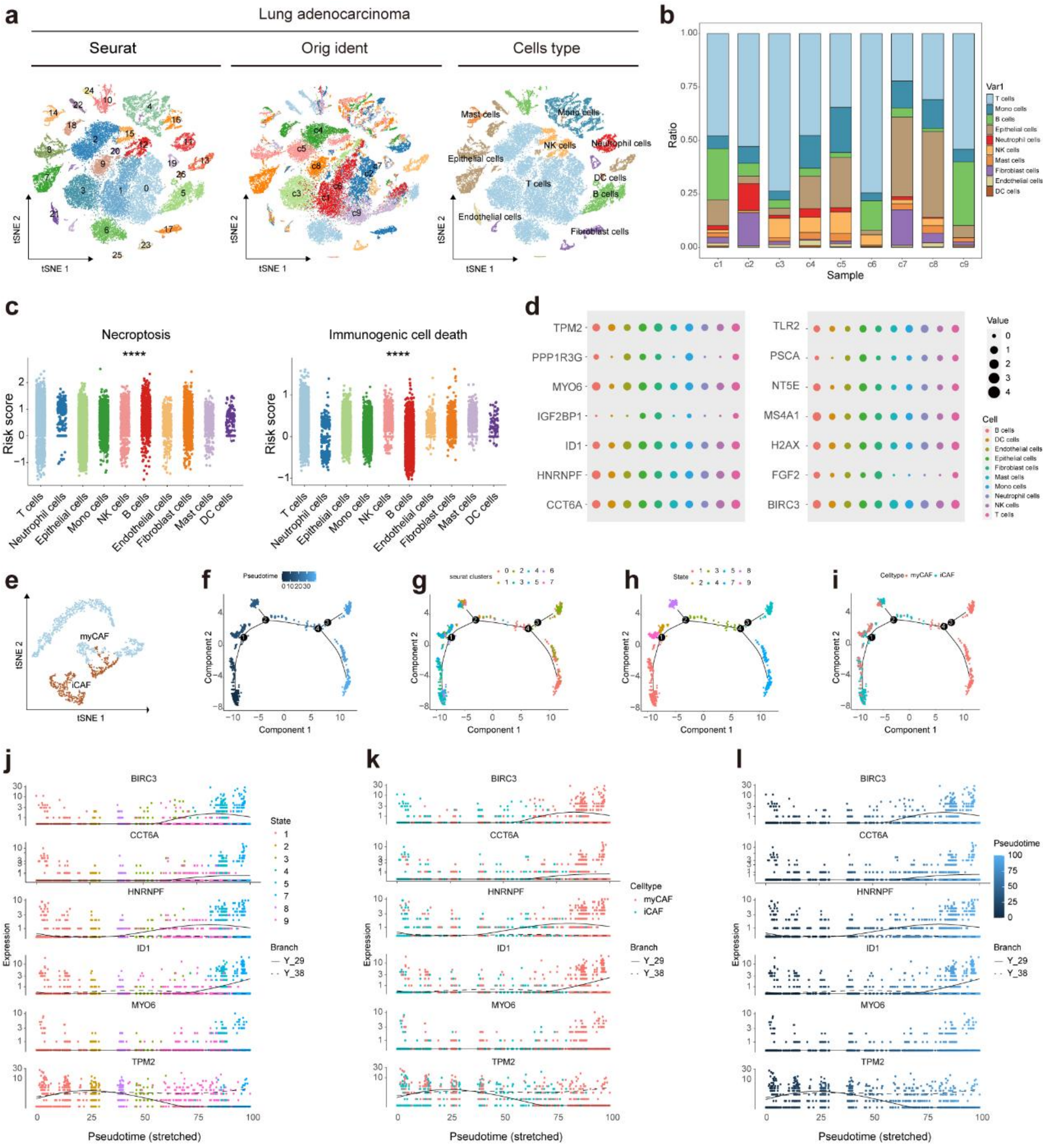

Supplementary Fig. 4 Intra-tumoral cellular heterogeneity of LUAD samples. a t-SNE plot showing the identified 10 cell types. **b** Fraction of cell types originating from each sample. **c** The risk score of necroptosis and ICD in different cell types, respectively. **d** Bubble plot of expression value of necroptosis and ICD related model genes in different cell subtypes, respectively. **e-i** Developmental trajectory of CAF cells inferred by monocle, colored by pseudotime, Seurat clusters, state, and cell subtype. **j-l** The relationship of expression in BIRC3, CCT6A, HNRNPF, ID1, MYO6 and TPM2 with the pseudotime colored by state, cell subtype and pseudotime, respectively. **P*<0.05, ***P*<0.01, ****P*<0.001.

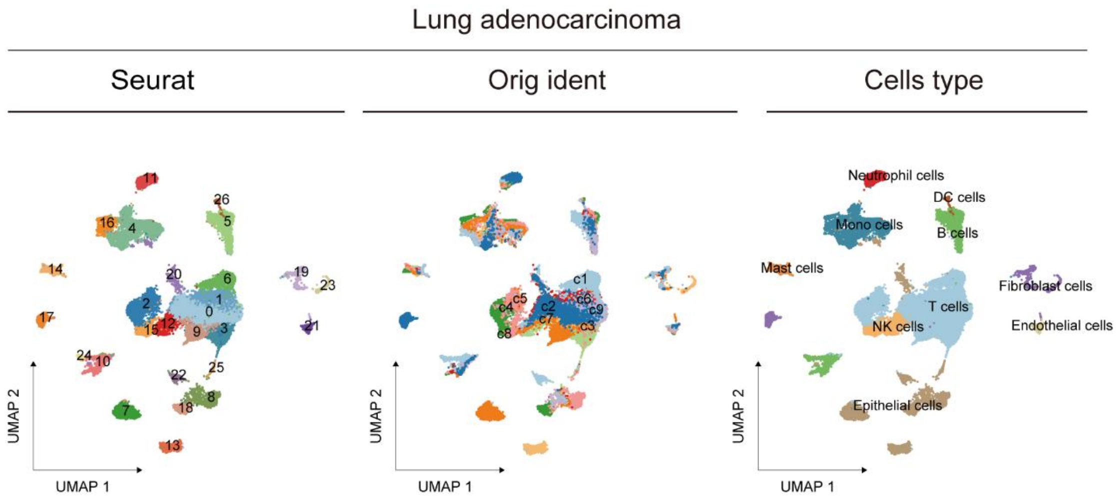

Supplementary Fig. 5 The UMAP plot visualization of 10 cell subtypes from 9 tumor tissue samples.

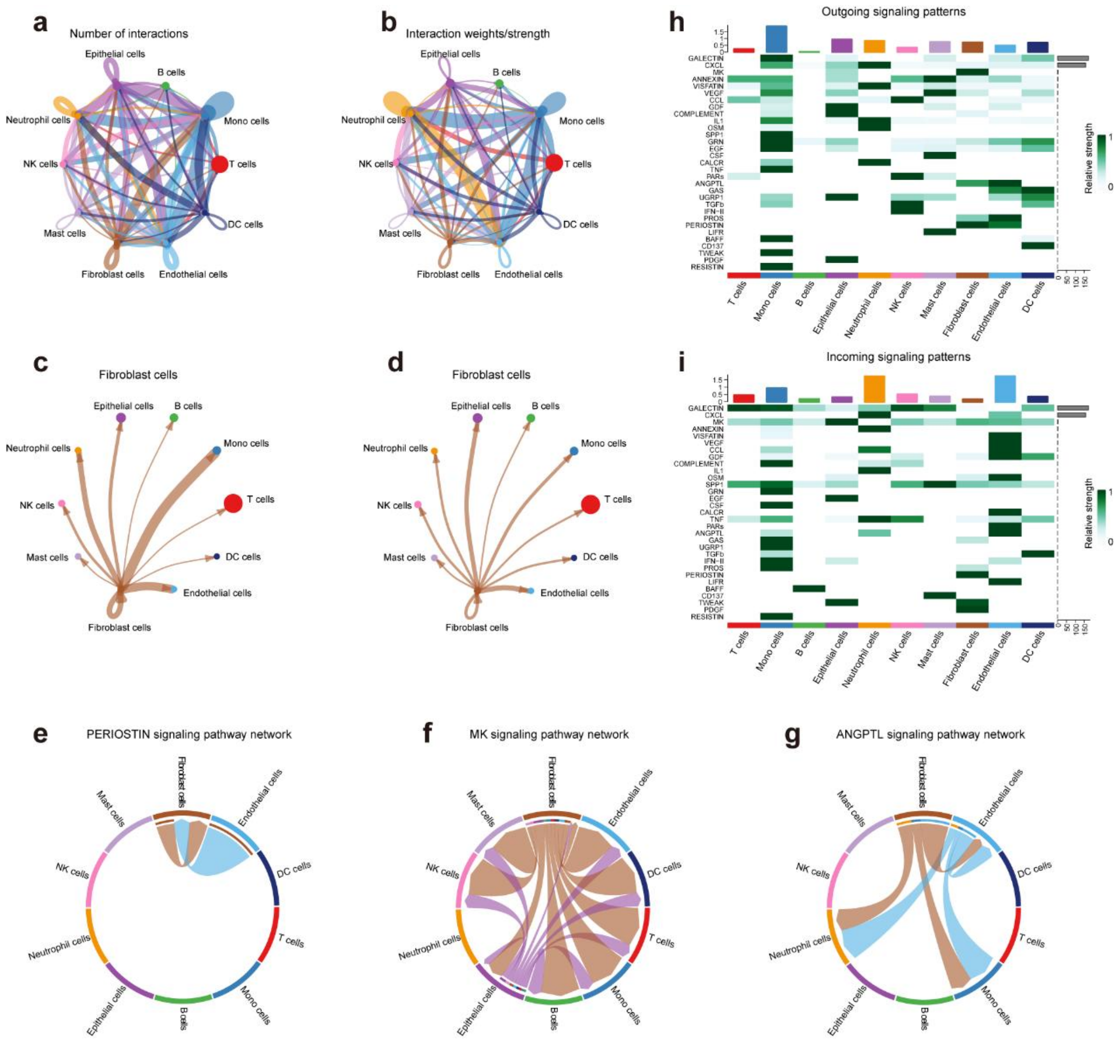

Supplementary Fig. 6 Cell communication network analysis in LUAD. a Chord plot showing the number of interactions among immune cell types. **b** Chord plot showing the interaction weights/strength among immune cell types. **c** The interaction number of CAF cells with other immune cell types. **d** The interaction weights/strength of CAF cells with other immune cell types. **e-g** Chart showing inferred intercellular communication network of PERIOSTIN, MIK and ANGPTL signaling pathway with immune cell types using autocrine and paracrine signaling. Bar sizes are proportional to the number of cells in each cell group and line width represents the communication probability. **h-i** The contribution weight of outgoing and incoming signaling patterns to immune cell types in cell communication network system.

Supplementary File. 1 The relationship of other cell death types related genes with OS in LUAD.

Anoikis related genes DAPK2 (HR=0.788, 95%CI=0.644-0.965, P=0.021), SNAI2 (HR=1.235, 95%CI=1.076-1.417, P=0.003), TLE1 (HR=1.463, 95%CI=1.169-1.830, P=0.001); apoptosis like morphology related genes APOL1 (HR=1.175, 95%CI=1.016-1.358, P=0.030), BCL2L1 (HR=1.345, 95%CI=1.045-1.733, P=0.022), PRKCD (HR=0.680, 95%CI=0.536-0.863, P=0.002), VDAC1 (HR=1.481, 95%CI=1.093-2.005, P=0.011); autophagy related genes APOL1 (HR=1.125, 95%CI=0.970-1.304, P=0.119), ATG12 (HR=1.572, 95%CI=1.056-2.342, P=0.026), CAPNS1 (HR=1.334, 95%CI=0.964-1.846, P=0.082), CCR2 (HR=0.714, 95%CI=0.608-0.840, P=4.66E-05), ITGA6 (HR=1.181, 95%CI=1.028-1.357, P=0.019), SPHK1 (HR=1.216, 95%CI=1.057-1.400, P=0.006); cuproptosis related genes LOXL2 (HR=1.288, 95%CI=1.119-1.481, P=0.0004), MT2A (HR=1.112, 95%CI=0.976-1.266, P=0.110), SLC25A3 (HR=1.339, 95%CI=0.935-1.916, P=0.111), SOD1 (HR=1.570, 95%CI=1.167-2.112, P=0.003); extrinsic apoptosis related genes AVEN (HR=1.260, 95%CI=0.926-1.714, P=0.142), F2 (HR=1.177, 95%CI=1.034-1.340, P=0.014), FGF2 (HR=1.360, 95%CI=1.092-1.692, P=0.006), H2AX (HR=1.351, 95%CI=1.095-1.668, P=0.005), PPP1R13B (HR= 0.840, 95%CI=0.678-1.041, P=0.111), RAC1 (HR=1.399, 95%CI=0.962-2.033, P=0.079), SHC1 (HR=1.315, 95%CI=1.005-1.720, P=0.046); ferroptosis related genes ACSL3 (HR=1.332, 95%CI=1.081-1.641, P=0.007), BRDT (HR=0.881, 95%CI=0.791-0.980, P=0.020), CDCA3 (HR=1.317, 95%CI=1.135-1.530, P=0.0003), DDIT4 (HR=1.251, 95%CI=1.091-1.434, P=0.001), DECR1 (HR=1.298, 95%CI=0.973-1.732, P=0.076); intrinsic apoptosis related genes F2 (HR=1.188, 95%CI=1.042-1.354, P=0.010), FGF2 (HR=1.366, 95%CI=1.091-1.709, P=0.007), FLNC (HR=1.105, 95%CI=0.987-1.238, P=0.083), H2AX (HR=1.274, 95%CI=1.017-1.594, P=0.035), PPIA (HR=1.575, 95%CI=1.106-2.243, P=0.012), PPP1R13L (HR=1.138, 95%CI=0.955-1.357, P=0.148), SHC1 (HR=1.280, 95%CI=0.981-1.671, P=0.069), TCN1 (HR=1.093, 95%CI=1.031-1.160, P=0.003); lysosome dependent cell death related genes CSNK1E (HR=1.350, 95%CI=1.056-1.727, P=0.017), CTSL (HR=1.293, 95%CI=1.098-1.522, P=0.002), MCOLN2 (HR=0.708, 95%CI=0.590-0.849, P=0.0002), PEBP1 (HR=0.797, 95%CI=0.602-1.056, P=0.114), PPIA (HR=1.604, 95%CI=1.131-2.274, P=0.008), RAB21 (HR=1.361, 95%CI=0.996-1.860, P=0.053), SBF2 (HR=1.246, 95%CI=0.993-1.564, P=0.058); necrosis like morphology related genes CASP9 (HR=1.294, 95%CI=1.003-1.670, P=0.047), CYCS (HR=1.106, 95%CI=0.825-1.482, P=0.500), GAPDH (HR=1.299, 95%CI=0.977-1.728, P=0.072), RAC1 (HR=1.427, 95%CI=0.964-2.111, P=0.075), SLC2A1 (HR=1.019, 95%CI=0.859-1.209, P=0.829), TYMS (HR=1.124, 95%CI=0.933-1.356, P=0.219); necrosis related genes C1QTNF6 (HR=1.243, 95%CI=1.040-1.485, P=0.017), F2 (HR=1.149, 95%CI=1.011-1.306, P=0.034), FGF2(HR=1.426, 95%CI=1.160-1.752, P=0.0007), GAPDH (HR=1.259, 95%CI=1.003-1.581, P=0.047), KRT18 (HR=1.190, 95%CI=0.975-1.452, P=0.087); parthanatos related genes FEN1 (HR=1.358, 95%CI=1.114-1.655, P=0.002), MCL1 (HR=1.312, 95%CI=1.001-1.721, P=0.049); pyroptosis related genes APOL1

(HR=1.195, 95%CI=1.040-1.373, P=0.012), CRTAC1(HR=0.920, 95%CI=0.833-1.016, P=0.098), CYCS (HR=1.309, 95%CI=1.008-1.698, P=0.043), EPHA2 (HR=1.189, 95%CI=1.033-1.368, P=0.016), H2AX (HR=1.250, 95%CI=1.001-1.561, P=0.049); entotic cell death related genes MRTFA (HR=1.471, 95%CI=1.095-1.977, P=0.010).

Supplementary File. 2 Formulas for risk scores related to other cell death types.

The anoikis related risk score = SNAI2*0.210994473 + TLE1*0.380249267 − DAPK2*0.237728624.

The apoptosis like morphology related score = APOL1*0.161101228 + BCL2L1*0.296690637 − PRKCD*0.38521057 + VDAC1*0.39250681.

The autophagy related score = APOL1*0.117631006 + ATG12*0.452845553 + CAPNS1*0.28803844 − CCR2*0.336416095 + ITGA6*0.166218778 + SPHK1*0.195793779.

The cuproptosis related risk score = LOXL2*0.25277854 + MT2A *0.106172376 + SLC25A3*0.29170742 + SOD1*0.451330863.

The extrinsic apoptosis related score = AVEN*0.230820981 + F2*0.163120003 + FGF2*0.307049536 + H2AX*0.301212678 − PPP1R13B*0.17425036 + RAC1*0.335574621 + SHC1*0.273838229.

The ferroptosis related score = ACSL3*0.286493196 − BRDT*0.127193832 + CDCA3*0.275700387 + DDIT4*0.223763467 + DECR1*0.260855506.

The intrinsic apoptosis related score = F2*0.172222435 + FGF2*0.311591781 + FLNC*0.100082114 + H2AX*0.241981665 + PPIA*0.454032076 + PPP1R13L*0.129568083 + SHC1*0.24698089 + TCN1*0.089202482.

The lysosome dependent cell death related score = CSNK1E*0.300192534 + CTSL*0.256908149 − MCOLN2*0.34589256 − PEBP1*0.226422736 + PPIA*0.472345756 + RAB21*0.308106581 + SBF2*0.220119029.

The necrosis like morphology related score = CASP9*0.257748389 + CYCS*0.100567382 + GAPDH*0.261679559 + RAC1*0.355311119 + SLC2A1*0.01881725 + TYMS*0.117308318.

The necrosis related score = C1QTNF6*0.21741645 + F2*0.138881517 + FGF2*0.354641587 + GAPDH*0.230658607 + KRT18*0.17383862.

The parthanatos related score = FEN1*0.306 + MCL1*0.272.

The pyroptosis related score = APOL1*0.178241005 − CRTAC1*0.083769597 + CYCS*0.269048127 + EPHA2*0.172708798 + H2AX*0.223048741.

The entotic cell death related score = MRTFA*0.386.
