# Supplementary material for: Implications of different cell death patterns for prognosis and immunity in lung adenocarcinoma
Source: NPJ Precis Oncol. 2023 Nov 15;7:121. doi: 10.1038/s41698-023-00456-y (PMC10651893; doi:10.1038/s41698-023-00456-y)
Supplement: Supplementary file 4 — REPORTING SUMMARY [file 41698_2023_456_MOESM4_ESM.pdf]

nature portfolio

Corresponding author(s): DBPR NPJPRECISIONONCOLOGY-03182R

Last updated by author(s): May 18, 2023

Reporting Summary

Nature Portfolio wishes to improve the reproducibility of the work that we publish. This form provides structure for consistency and transparency in reporting. For further information on Nature Portfolio policies, see our Editorial Policies and the Editorial Policy Checklist.

Statistics

For all statistical analyses, confirm that the following items are present in the figure legend, table legend, main text, or Methods section.

n/a	Confirmed	
☐	☒	The exact sample size (*n*) for each experimental group/condition, given as a discrete number and unit of measurement
☐	☒	A statement on whether measurements were taken from distinct samples or whether the same sample was measured repeatedly
☐	☒	The statistical test(s) used AND whether they are one- or two-sided<br>*Only common tests should be described solely by name; describe more complex techniques in the Methods section.*
☒	☐	A description of all covariates tested
☒	☐	A description of any assumptions or corrections, such as tests of normality and adjustment for multiple comparisons
☐	☒	A full description of the statistical parameters including central tendency (e.g. means) or other basic estimates (e.g. regression coefficient) AND variation (e.g. standard deviation) or associated estimates of uncertainty (e.g. confidence intervals)
☐	☒	For null hypothesis testing, the test statistic (e.g. *F*, *t*, *r*) with confidence intervals, effect sizes, degrees of freedom and *P* value noted<br>*Give P values as exact values whenever suitable.*
☒	☐	For Bayesian analysis, information on the choice of priors and Markov chain Monte Carlo settings
☒	☐	For hierarchical and complex designs, identification of the appropriate level for tests and full reporting of outcomes
☒	☐	Estimates of effect sizes (e.g. Cohen's *d*, Pearson's *r*), indicating how they were calculated

Our web collection on statistics for biologists contains articles on many of the points above.

Software and code

Policy information about availability of computer code

Data collection	We selected the key regulatory genes for 15 cell death patterns including pyroptosis, ferroptosis, necroptosis, autophagy, immunologic cell death, entotic cell death genes, cuproptosis, parthanatos, lysosome-dependent cell death, intrinsic apoptosis, extrinsic apoptosis, necrosis, anoikis, apoptosis-like morphology and necrosis-like morphology using a combination of Gene Set Enrichment Analyses (GSEA) gene sets from MSigDB (http://software.broadinstitute.org/gsea/msigdb/index.jsp), Kyoto Encyclopedia of Genes and Genomes(KEGG), review articles, and manual collection of gene sets from Genecards website (https://www.genecards.org/). GeneCards is an integrative human gene database in which there is highly comprehensive and user-friendly gene information to help us better investigate human genetic research.
Data analysis	R version 4.1.3 was used for all statistical studies. The survival curve was plotted by Kaplan-Meier survival curve. The student's t-test and Wilcoxon test were used to compare the differences between two groups, and Spearman analysis was used to calculate the correlation coefficients.

For manuscripts utilizing custom algorithms or software that are central to the research but not yet described in published literature, software must be made available to editors and reviewers. We strongly encourage code deposition in a community repository (e.g. GitHub). See the Nature Portfolio guidelines for submitting code & software for further information.

Data

Policy information about availability of data

All manuscripts must include a data availability statement. This statement should provide the following information, where applicable:
- Accession codes, unique identifiers, or web links for publicly available datasets
- A description of any restrictions on data availability
- For clinical datasets or third party data, please ensure that the statement adheres to our policy

The data used to support the findings of this study are available from the corresponding author upon request.

Research involving human participants, their data, or biological material

Policy information about studies with human participants or human data. See also policy information about sex, gender (identity/presentation), and sexual orientation and race, ethnicity and racism.

Reporting on sex and gender	Not applicable
Reporting on race, ethnicity, or other socially relevant groupings	Not applicable
Population characteristics	See above
Recruitment	We collected fresh tissue specimens from 5 patients with LUAD receiving surgical operation in the Harbin medical university cancer hospital. No information was collected from patients for statistical analysis.
Ethics oversight	Not applicable

Note that full information on the approval of the study protocol must also be provided in the manuscript.

Field-specific reporting

Please select the one below that is the best fit for your research. If you are not sure, read the appropriate sections before making your selection.

☒ Life sciences ☐ Behavioural & social sciences ☐ Ecological, evolutionary & environmental sciences

For a reference copy of the document with all sections, see nature.com/documents/nr-reporting-summary-flat.pdf

Life sciences study design

All studies must disclose on these points even when the disclosure is negative.

Sample size	Raw bulk transcriptome counts, normalized and log2 converted RNA-sequencing profiles FPKM as well as clinical information for LUAD patients and normal samples were identified from the University of California Santa Cruz (UCSC) database. We annotated the genes using the Gencode (GENCODE.v32) (ftp://ftp.ebi.ac.uk/pub/databases/gencode/Gencode_human/) GTF file and then normalized. To convert ensemble ids into gene symbols, the "GeoTcgaData" R package was applied. We also applied GSE171145 including 40,799 single cells from 9 LUAD samples to obtain single-cell RNA transcriptome data for LUAD patients, and the “Seurat V4” R package was applied for analysis.
Data exclusions	We have excluded samples with incomplete clinical information from the database.
Replication	The reagents and materials used in the experiments are described in detail, as well as the specific experimental procedures.
Randomization	We downloaded samples as training set in TCGA database. GSE37745 and GSE68465 in GEO database were used as validation sets. GSE135222, GSE78220 and IMvigor 210 cohort were also applied to assess the response to immunotherapy between high and low CDI group. Besides, GSE171145 including 40,799 single cells from 9 LUAD samples was downloaded to obtain single-cell RNA transcriptome data.
Blinding	The data for this study were obtained from public databases and did not require researchers to collect. Therefore blinding was not relevant to this study.

Reporting for specific materials, systems and methods

We require information from authors about some types of materials, experimental systems and methods used in many studies. Here, indicate whether each material, system or method listed is relevant to your study. If you are not sure if a list item applies to your research, read the appropriate section before selecting a response.

Materials & experimental systems

n/a	Involved in the study
☐	☒ Antibodies
☐	☒ Eukaryotic cell lines
☒	☐ Palaeontology and archaeology
☒	☐ Animals and other organisms
☒	☐ Clinical data
☒	☐ Dual use research of concern
☒	☐ Plants

Methods

n/a	Involved in the study
☒	☐ ChIP-seq
☒	☐ Flow cytometry
☒	☐ MRI-based neuroimaging

Antibodies

Antibodies used	HNRNPF (Affinity Biosciences Cat# DF7383, RRID:AB_2839321), FGF2 (Affinity Biosciences Cat# DF6038, RRID:AB_2838011), MLKL (Affinity Biosciences Cat# DF7412, RRID:AB_2839350), Phospho-MLKL (Affinity Biosciences Cat# AF7420, RRID:AB_2843860), eIF2α (Affinity Biosciences Cat# AF6087, RRID:AB_2834856), Phospho-eIF2α (Affinity Biosciences Cat# AF3087, RRID:AB_2834524), β-actin (Beijing Zhongshan Golden Bridge Biotechnology Co.Ltd Cat# TA-09, clone name: OTI1), and GAPDH (Absin Cat# abs830030, clone name: GA12)
Validation	HNRNPF antibody was used for Western blot, with a dilution ratio of 1:1000; FGF2 antibody was used for Western blot, with a dilution ratio of 1:1000;MLKL antibody was used for Western blot, with a dilution ratio of 1:1000; Phospho-MLKL antibody was used for Western blot, with a dilution ratio of 1:1000; eIF2α antibody was used for Western blot, with a dilution ratio of 1:1000; Phospho-eIF2α antibody was used for Western blot, with a dilution ratio of 1:1000; β-actin antibody was used for Western blot, with a dilution ratio of 1:1000; GAPDH antibody was used for Western blot, with a dilution ratio of 1:1000.

Eukaryotic cell lines

Policy information about cell lines and Sex and Gender in Research

Cell line source(s)	The Human bronchial epithelial cells (HBE) and a collection of lung cancer cells (A549, NCI-H1299, NCI-H1915, and H1650) were obtained from Laboratory of Medical Genetics (Department of Biology, Harbin Medical University, Harbin, China).
Authentication	The cell lines used in this study are all authenticated. The authentication techniques include cell morphological examination, growth curve analysis and isozyme analysis.
Mycoplasma contamination	All cell lines tested negative for mycoplasma contamination
Commonly misidentified lines (See ICLAC register)	Not applicable
